# Transcriptome Transformer: improving patient survival prediction via multitask learning of transcriptomic and clinical features

**DOI:** 10.1093/bib/bbaf628

**Published:** 2025-11-25

**Authors:** Bonil Koo, Inyoung Sung, Sangseon Lee, Sun Kim

**Affiliations:** Interdisciplinary Program in Bioinformatics, Seoul National University, 1, Gwanak-ro, 08826 Seoul, Republic of Korea; AIGENDRUG Co., Ltd., 1793, Nambusunhwan-ro, 08758 Seoul, Republic of Korea; BK21 FOUR Intelligence Computing, Seoul National University, 1, Gwanak-ro, 08826 Seoul, Republic of Korea; Department of Artificial Intelligence, Inha University, 100 Inha-ro, 22180 Incheon, Republic of Korea; Interdisciplinary Program in Bioinformatics, Seoul National University, 1, Gwanak-ro, 08826 Seoul, Republic of Korea; AIGENDRUG Co., Ltd., 1793, Nambusunhwan-ro, 08758 Seoul, Republic of Korea; Department of Computer Science and Engineering, Seoul National University, 1, Gwanak-ro, 08826 Seoul, Republic of Korea; Interdisciplinary Program in Artificial Intelligence, Seoul National University, 1, Gwanak-ro, 08826 Seoul, Republic of Korea

**Keywords:** transcriptome, transformer, survival prediction, multitask learning, gene expression

## Abstract

Accurate survival prediction is essential in healthcare as it guides treatment strategies and improves patient outcomes. While clinical features provide valuable prognostic information, they often fail to represent the molecular complexity of diseases. Transcriptomic data, which reflects gene expression patterns of tumors, present a complementary perspective to address this limitation. We introduce *Transcriptome Transformer* (TxT), a multitask learning framework that uses a transcriptome-centric approach to improve patient survival prediction. TxT employs a Transformer-based architecture with multihead attention mechanisms to effectively capture complex dependencies among genes, enabling dynamic modeling of gene–gene interactions while using shared information across multiple clinical prediction tasks. By jointly analyzing transcriptomic data and incorporating clinical features, TxT offers a more complete representation of patient biology. In experiments across both single-task and multitask datasets, TxT outperformed existing methods in survival prediction and related clinical tasks. Additionally, TxT offers biological insights through attention-derived gene interaction networks, identifying immune-related pathways in longer-surviving Luminal A patients and coagulation and epithelial–mesenchymal transition pathways in shorter-surviving counterparts. Differential attention analysis further revealed that integrating clinical features enhances the model’s ability to prioritize genes involved in biologically meaningful pathways that are known to influence tumor progression and distant recurrence. The source code of TxT is available at https://github.com/BonilKoo/TxT.

## Introduction

### Motivation

Accurate prediction of survival outcomes in cancer patients is crucial for guiding personalized treatment decisions, optimizing resource allocation, and informing patients about their prognosis [1]. While traditional clinical features, such as age and tumor size, are important predictors of survival and recurrence, they are not designed to probe the molecular complexity and heterogeneity of cancer that can significantly influence patient outcomes [2].

Advances in transcriptomic profiling have revealed that gene expression patterns offer critical insights into tumor biology, disease progression, and patient outcomes [3]. For instance, overexpression of osteopontin (*SPP1*) has been shown to upregulate key epithelial–mesenchymal transition (EMT) transcription factors, thereby promoting aggressive phenotypes in breast cancer [4]. In triple-negative breast cancer, elevated expression of immune-related genes is associated with better survival, suggesting that immune transcriptomic activity can outweigh otherwise aggressive tumor biology [5]. EMT-related expression programs have also been consistently linked to recurrence and therapeutic resistance across various tumor types [6]. These findings underscore the clinical relevance of transcriptomic data and have led to an increased focus on leveraging gene expression profiles to infer clinical features and improve survival prediction in cancer.

Among clinical factors, age and tumor size have long been recognized as significant predictors of survival and recurrence in cancer patients. However, leveraging additional molecular data, particularly transcriptomics, to augment the predictive power of these established factors continues to be a significant research focus. While previous studies have explored the integration of genomic mutations with clinical features [7], approaches that employ a transcriptome-centric strategy to infer clinical features and predict survival are not well studied.

**Figure 1:**
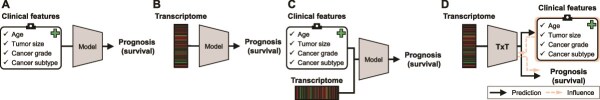
Approaches to survival prediction using clinical and transcriptomic data. (A) A model that predicts survival outcomes based solely on clinical features. (B) A model that relies exclusively on transcriptome-wide gene expression data for survival prediction. (C) A model that combines both clinical features and transcriptomic data to predict patient prognosis, leveraging complementary information from these data sources. (D) The proposed *Transcriptome Transformer* (TxT) framework, which employs multitask learning (MTL) to jointly integrate transcriptomic and clinical features, leading to enhanced survival prediction and clinical-feature inference.

In this study, we propose a transcriptome-centric approach to survival prediction. Our goal is to leverage the rich information in transcriptomic data together with clinical features for patient survival prediction so that survival prediction accuracy can be improved in explainable ways in terms of molecular mechanisms of cancers (Fig. 1).

### Challenge

In developing a transcriptome-centric model for patient survival prediction, two main challenges arise. First, transcriptomic profiles are real-valued high-dimensional vectors representing expression levels across numerous genes while clinical features are often single values (e.g. patient age or cancer grade). Designing a prediction model that primarily processes transcriptomic data while effectively incorporating clinical features presents significant challenges, as each data type must be handled in a way that preserves its unique properties and maximizes its contribution to the prediction task.

Second, predicting survival from transcriptomic data requires representing intricate gene interactions. A single gene may carry out multiple functions depending on the regulatory setting. For example, p53 is involved in DNA repair, cell-cycle arrest, and apoptosis [8, 9]. Considering these varied functions requires a modeling strategy that can represent dynamic interactions among genes. Understanding these interactions is crucial for survival prediction because they can reveal potential therapeutic targets or biomarkers associated with disease progression. Moreover, the technical challenges of unifying clinical and transcriptomic data continue to be substantial, given their different scales and data structures. Resolving these issues in a single framework may enhance the predictive capability for both established clinical features and complex molecular indicators.

### Approach

This study introduces TxT, an MTL model that adopts a transcriptome-centric approach to patient survival prediction. TxT primarily processes transcriptomic data while integrating clinical features in a complementary manner, addressing the challenges of handling disparate data types, and modeling complex gene–gene interactions. The model employs a Transformer-based architecture [10], applying multihead attention to capture complex relationships among genes, which is essential for accurately modeling the intricate biological processes involved in cancer. This approach is based on the principle that each gene’s role may vary depending on its regulatory contexts, similar to how a word’s interpretation can shift depending on its surrounding text.

TxT utilizes a novel positional embedding method that interprets gene expression levels as position-like information, allowing the model to create patient-specific representations based on individual molecular profiles. Traditional positional encoding methods impose fixed or artificially defined positional rules, which may not properly represent the dynamic and context-dependent interactions among genes. Instead, TxT interprets gene expression levels as position-like information, enabling the creation of patient-specific representations grounded in each individual’s molecular profile. This data-driven approach offers greater flexibility, allowing the model to adaptively capture complex gene interactions and better represent the unique regulatory landscapes present in different patients.

To implement this transcriptome-centric approach, TxT utilizes an MTL framework that simultaneously predicts multiple clinical features and survival outcomes from transcriptomic data. By formulating survival prediction tasks in a unified framework, the model leverages the mutually beneficial connections between tasks, where improvements in predicting clinical features can enhance survival prediction accuracy. Such an approach enables the model to learn reliable data patterns that capture both established prognostic markers and high-dimensional transcriptomic signals. Ultimately, these shared representations enhance survival prediction performance, which is the primary focus of this work.

## Related work

### Transformer

While Transformer-based architectures have been widely studied in various fields, their application to bulk RNA-seq transcriptome data remains relatively underexplored. Transformer is designed to work with sequences of tokens, but transcriptome data usually comes in the form of numerical values (i.e. gene expression quantities). DeepGene Transformer [11] processes gene expression levels as a 1D vector, using convolutional neural networks (CNNs) instead of positional encoding for the multihead attention layer. However, this CNN-based approach may limit the model’s ability to learn explicit relationships between genes in the multihead attention layer.

T-GEM [12] uses gene expression quantities directly as tokens and employs gene-dependent parameters, distinguishing it from traditional Transformers. While this approach allows for gene-specific learning, it increases the number of model parameters proportionally to the number of genes used. Additionally, T-GEM’s learning is limited to the context provided by transcriptome data without incorporating prior biological knowledge.

### Multitask learning

MTL is an approach that aims to improve model performance by jointly learning multiple related tasks [13, 14]. This technique can lead to more generalized and robust data representations, which is particularly valuable in complex biological contexts. MTL is widely studied in various fields, such as computer vision [15, 16], natural language processing (NLP) [17, 18], and drug discovery [19, 20]. Recently, studies related to MTL have also been conducted in the field of bioinformatics [21, 22].

OmiEmbed [23], to our knowledge, is the only study that uses omics data to simultaneously predict age, cancer type, and survival. While it uses a variational autoencoder (VAE) [24] architecture and GradNorm [25] optimization, the VAE’s constraint to specific distributions may limit its flexibility in modeling complex gene interactions. Our approach aims to address this limitation through a more flexible Transformer-based architecture.

## Materials and methods

### Computational formulation of transcriptome data for transformer models

To effectively utilize transcriptome data within the Transformer framework, we adopt a formulation inspired by NLP. In the case of texts, words in a sentence are vectorized using the word embedding method [26], and the position of each word in the sentence is encoded using positional encoding [10, 27]. As a result, the words and position information within the sentence collectively learn the context of each word. Subsequently, the word embeddings with position information are used as input to the Transformer for performing specific tasks such as text classification.

In the context of transcriptome data, we liken a patient’s transcriptomic profile to a sentence, where individual genes correspond to words. This analogy helps illustrate how understanding the relationship between genes and their expression levels enables the model to learn the context of genes within each patient. Consequently, both embedding and positional information of genes are integrated to form comprehensive representations. Specifically, transcriptome data, such as gene expression levels, provide position-like information that updates gene embeddings for each sample through the layers of the transformer. This novel approach enhances the model’s ability to capture intricate gene–gene interactions and predict patient conditions or outcomes effectively. Importantly, we do not assume or impose any biologically meaningful ordering of genes. Gene expression values are treated as an unordered set, and any positional information is learned in a data-driven manner through transcriptome-based embeddings.

### Model architecture

**Figure 2:**
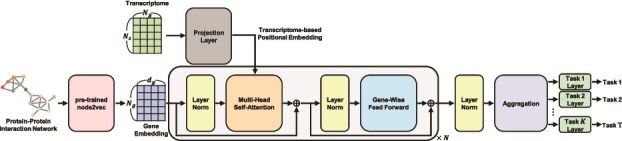
Illustration of the proposed model architecture. The embedding for each gene, used as input to the transformer, is derived from a pretrained node2vec model on the protein–protein interaction (PPI) network. Gene expression values are transformed through a projection layer, serving as transcriptome-based positional embedding. These embeddings are processed through multihead self-attention and gene-wise feed-forward layers to capture complex gene–gene interactions and sample-specific transcriptomic profiles. Finally, the latent embeddings are passed to task-specific layers to predict outcomes for multiple tasks. Notations: $N_{s}$ denotes the number of samples, $N_{g}$ represents the number of genes, $d_{g}$ is the embedding dimension of a gene, $N$ signifies the number of encoder layers, and $T$ represents the number of tasks.

Building upon the computational formulation, we present the architecture of the TxT model, which is designed to leverage both transcriptomic and clinical data for improved survival prediction. The overview of the model architecture is shown in Fig. 2.

#### Pretraining gene embeddings on biological networks

Genes interact with each other and function collectively in complexes, while certain genes are co-expressed due to a shared regulatory mechanism. A biological network serves as a representation of the intricate interactions and relationships among various biological entities within a living system. Among various types of biological networks, the protein–protein interaction (PPI) network represents the physical or functional associations between proteins or genes and is widely used to encode biologically relevant gene–gene dependencies in transcriptomic modeling studies [28, 29].

Instead of employing randomly initialized gene embeddings as input to the Transformer, we effectively utilize pretrained gene embeddings on the PPI network to incorporate informative prior knowledge. Among gene embedding methods, we used node2vec [30] to encode biological context into the gene representations. This initialization provides the model with inductive biases that reflect known biological structure, potentially improving generalization, and interpretability in downstream predictive tasks.

#### Transcriptome-based positional embedding

As discussed in Section “Computational formulation of transcriptome data for transformer models”, transcriptome data play a pivotal role akin to positional encoding for gene embeddings in each sample. Unlike traditional positional encodings used for sequence data, our approach does not rely on any predefined ordering of genes and instead learns position-related signals directly from expression profiles.

**Figure 3:**
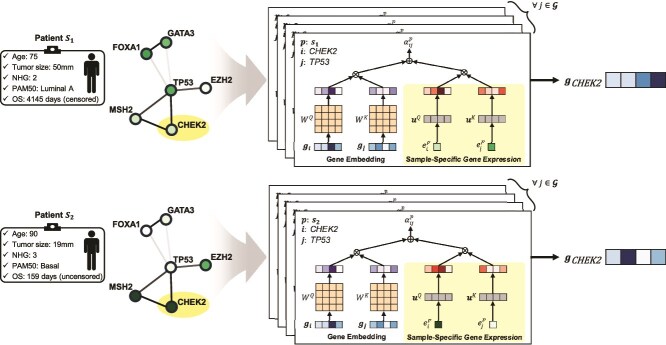
Transcriptome-based positional embedding. The transcriptomic data serve as a position-like role, enabling sample-specific updates to gene embeddings across transformer layers. Initially, all gene embeddings are equally initialized, but they are dynamically adjusted based on patient-specific transcriptomic profiles during model processing. Notations: $\mathbf{g}_{i}$ represents the embedding of gene $i$, $W^{Q}$ and $W^{K}$ denote the learnable parameters for query and key in the multihead attention layer, respectively, $e_{i}^{p}$ signifies the gene expression value for gene $i$ of patient $p$, $\mathbf{u}^{Q}$ and $\mathbf{u}^{K}$ represent learnable parameters for query and key in the projection layer, respectively,$\alpha _{ij}^{p}$ denotes the attention value for the query gene $i$ and key gene $j$ pair of sample $p$, and $\mathcal{G}$ represents the gene set used.

Gene embeddings and transcriptome data originate from distinct heterogeneous resources. Drawing inspiration from TUPE [31], which is an effective alteration that unties words and positional information in the Transformer, we introduce projection layers for our novel approach called *transcriptome-based positional embedding* (Fig. 3). This process occurs during the calculation of attention values ($\alpha ^{p}_{ij}$) in the attention layers, specifically for each query gene $i$ and key gene $j$ pair of sample $p$, as defined by the equation: 


(1) \begin{align*}& \alpha^{p}_{ij} = \frac{1}{\sqrt{2d}}(\mathbf{g}^{l}_{i} W^{Q,l})(\mathbf{g}^{l}_{j} W^{K,l})^\intercal + \frac{1}{\sqrt{2d}}(e^{p}_{i} \mathbf{u}^{Q})(e^{p}_{j} \mathbf{u}^{K})^\intercal.\end{align*}

Here, $d$ represents the dimensionality of hidden representations, $\mathbf{g}^{l}_{i}$ is the embedding vector of gene $i$ in the $l$th layer, and $e^{p}_{i}$ denotes the gene expression value of gene $i$ for sample $p$. Matrices $W^{Q,l}$ and $W^{K,l}$ are projection matrices for the gene embedding of query and key genes in the $l$th layer, respectively, while vectors $\mathbf{u}^{Q}$ and $\mathbf{u}^{K}$ are projection vectors for the positional embedding of query and key genes. Consequently, by incorporating transcriptomic profile information into the initially consistent gene embeddings across all samples, it becomes possible to tailor the gene embedding specifically for each sample through the layers of the transformer, facilitating prediction of the target variable associated with that particular sample.

#### Multitask optimization technique

TxT employs an MTL framework to simultaneously predict multiple clinical features and survival outcomes by sharing transformer encoder layers across all tasks while maintaining task-specific output layers. Specifically, the shared encoder learns robust representations that capture both clinical and transcriptomic signals, which are then fed into separate heads tailored for each prediction task.

In the MTL, a model is typically trained to minimize a composite objective function represented by the equations: 


(2) \begin{align*}& \min_{\theta_{\text{sh}}, \theta_{1}, \cdots, \theta_{T}}\sum_{t=1}^{T}\lambda_{t}\hat{L_{t}}(\theta_{\text{sh}},\theta_{t}),\end{align*}

and 


(3) \begin{align*}& \hat{L_{t}}(\theta_{\text{sh}},\theta_{t})=\frac{1}{N}\sum_{p=1}^{N}L_{t}(f_{t}(\mathbf{x}^{p};\theta_{\text{sh}},\theta_{t}),y^{p}_{t}).\end{align*}

Here, $T$ denotes the number of tasks, $\lambda _{t}$ and $\hat{L_{t}}$ are coefficients and loss for task $t$, respectively, and $\theta _{\text{sh}}$ and $\theta _{t}$ are shared parameters and task-specific parameters of model $f_{t}$ for task $t$, respectively. $N$ represents the number of samples, and $\mathbf{x}^{p}$ and $y^{p}_{t}$ denote input features and the target variable for task $t$ of sample $p$, respectively. The loss function $L_{t}(\cdot ,\cdot )$ varies according to the task $t$, typically adopting mean squared error for regression and cross-entropy for classification. In our study on survival prediction using neural networks, we employed the loss function proposed by N-MTLR [32], which models survival as a sequence of binary classification tasks over discretized time intervals (Supplementary Methods).

To optimize MTL, various techniques have been developed. Some studies focus on finding optimal values of $\lambda _{t}$ to ensure effective training across all tasks [15, 25, 33]. In contrast, PCGrad [34] represents a distinct approach by minimizing conflicts among tasks and guiding the gradient vectors themselves so that all tasks learn in an appropriate direction, without relying on predefined or learned task-specific weights. If a gradient of one task exhibits negative cosine similarity to gradients of multiple tasks, the gradients are conflicting. Specifically, this becomes problematic when the gradient magnitude differences are large or there is a high curvature in the objective landscape. PCGrad addresses these issues by projecting the gradient vector of one task onto the normal plane of another task in the presence of conflicting gradient vectors. The projection is expressed as: 


(4) \begin{align*}& \mathbf{v}_{i} \leftarrow \mathbf{v}_{i} - \frac{\mathbf{v}_{i}\cdot \mathbf{v}_{j}}{\|\mathbf{v}_{j}\|^{2}}\mathbf{v}_{j},\end{align*}

where $\mathbf{v}_{i}$ represents the gradient vector of task $i$.

### Differential attention analysis

To assess how incorporating clinical features alters the distribution of gene-level attention weights, we trained two variants of our model—one with clinical outputs (multitask) and one without (single-task)—and compared their attention patterns. For each gene, we computed an aggregate attention score under both settings and performed a *t*-test to identify genes whose attention increased significantly with clinical features. We then applied false discovery rate (FDR) correction to account for multiple comparisons, retaining only those genes with adjusted *P*-values <.05. Finally, pathway enrichment analysis was conducted on the genes showing clinically influenced attention gains, offering insight into the molecular processes that become more prominent when clinical variables guide the model’s attention.

### Gene interaction modeling via multihead attention

After training TxT, we constructed a gene interaction network by extracting attention weights from multihead attention layers for each test sample. Specifically, if the attention weight between query and key genes within each head exceeds 0.01, an edge is formed from the key gene to the query gene. Combining these networks from all heads resulted in the comprehensive gene interaction network for each sample. Subsequently, for each sample, we identified genes with out-degree centrality surpassing $\mu _{\text{out}}+2\cdot \sigma _{\text{out}}$ as *hub genes*, while those with in-degree centrality exceeding $\mu _{\text{in}}+2\cdot \sigma _{\text{in}}$ were designated as *attractor genes*. Here, $\mu _{\text{out}}$ and $\sigma _{\text{out}}$ represent the average out-degree centrality and standard deviation of out-degree centrality in a network, respectively, and $\mu _{\text{in}}$ and $\sigma _{\text{in}}$ denote the average in-degree centrality and standard deviation of in-degree centrality in a network, respectively.

To facilitate the comparison and interpretation of hub and attractor genes between groups, the proportion of each gene selected in each group was computed. By counting the frequency with which each gene was selected in both groups and conducting the Fisher’s exact test, we obtained *P*-values indicating the differences in gene selection between the groups. Similar to the differentially expressed gene analysis, the ratio in the proportion of samples selecting each gene in each group was calculated. Genes with an absolute proportion ratio >2 and an adjusted *P*-value corrected by FDR <.05 were identified, thus revealing *core genes* for each group.

### Experiments

#### Datasets

In our experiments, we evaluated model performance on three different datasets, covering both single-task learning (STL) and MTL scenarios. For MTL, we used the SCAN-B dataset [35], focusing on breast cancer under two survival endpoints—overall survival (OS) and distant recurrence-free interval (DRFi). Additionally, the TARGET-AML dataset [36] was employed, where age and OS were framed as multitask objectives in a pediatric acute myeloid leukemia (AML) cohort. For STL, we used TCGA-BRCA [37] for PAM50 subtype classification in breast cancer patients. Further details about data selection and characteristics are described in Supplementary Methods.

#### Data preprocessing

We pretrained gene embeddings on the STRING (v12.0) network [38] using node2vec [30] and selected 1000 highly variable genes from MSigDB hallmark sets [39]. Log-transformed gene expression values (log$_{2}$(FPKM+1)) were normalized for each gene. A detailed description of sample selection, replicate handling, and thresholding is provided in Supplementary Methods.

#### Model training

Each dataset was randomly split into training (70%), validation (10%), and test (20%) sets 10 times to evaluate model robustness. We employed the Adam optimizer with a 0.0001 learning rate and applied early stopping based on validation loss to prevent overfitting. The best-performing model on the validation set was then used for the final evaluation. Additional model architecture details and hyperparameter tuning procedures are available in Supplementary Methods.

## Results

### Predictive performance across multiple datasets

We first assessed all competing methods on the SCAN-B dataset under two survival endpoints: OS and DRFi. In both scenarios, five tasks were jointly analyzed: predicting age, tumor size, Nottingham histological grade (NHG), PAM50, and survival (OS or DRFi). Table 1 and Table S7 summarize the results for OS and DRFi, respectively.

**Table 1:** Performance comparison on the SCAN-B OS dataset using various methods and evaluation metrics. Standard deviations and additional evaluation metrics are provided in Table S6. The best performance for each metric is highlighted in bold, and the second-best is underlined. Metrics with higher values indicating better performance are marked with ($\uparrow $), while those with lower values being better are marked with ($\downarrow $). OS, overall survival; NHG, Nottingham histological grade; C-Index, Concordance Index; IBS, Integrated Brier Score; ACC, accuracy; MAE, mean absolute error; SCC, Spearman’s correlation coefficient; SVM, support vector machine; RF, random forest; MLP, multilayer perceptron

Method		OS		PAM50		NHG		Age		Tumor size	
		C-Index ($\uparrow $)	IBS ($\downarrow $)	ACC ($\uparrow $)	F1 ($\uparrow $)	ACC ($\uparrow $)	F1 ($\uparrow $)	MAE ($\downarrow $)	SCC ($\uparrow $)	MAE ($\downarrow $)	SCC ($\uparrow $)
STL	SVM	0.645	–	0.905	0.865	0.719	0.623	8.420	0.599	6.965	0.480
	RF	0.639	0.092	0.882	0.821	0.698	0.585	8.593	0.553	7.764	0.393
	MLP	0.669	0.108	0.899	0.864	0.710	0.648	8.009	0.645	7.646	0.450
	OmiEmbed [23]	0.674	0.098	0.872	0.832	0.670	0.625	7.296	0.672	7.179	0.484
	T-GEM [12]	0.696	0.096	0.914	0.883	0.713	0.640	15.280	0.270	11.647	0.308
	Autosurv [40]	0.632	0.096	0.664	0.464	0.632	0.482	65.479	0.148	20.109	0.200
	SurvConvMixer [41]	0.672	0.100	0.848	0.792	0.686	0.569	9.105	0.461	7.714	0.345
	CNN+FMAP [42]	0.677	0.090	0.817	0.766	0.617	0.564	10.562	0.029	8.136	0.034
	TxT	0.701	0.087	**0.920**	**0.891**	0.715	0.649	7.185	**0.684**	6.778	0.485
MTL	OmiEmbed [23]	0.778	0.112	0.898	0.860	0.712	0.635	7.231	0.673	6.735	0.518
	TxT	**0.797**	**0.084**	**0.920**	**0.891**	**0.722**	**0.667**	**7.140**	0.681	**6.721**	**0.519**

On the SCAN-B OS dataset (Table 1), TxT (MTL) demonstrated better performance across nearly all metrics, including the lowest mean absolute error (MAE) for predicting age and tumor size, and the highest classification accuracy for NHG and PAM50. Moreover, TxT (MTL) achieved a C-Index of 0.797, compared to 0.701 under TxT (STL), corresponding to a 13.7% relative improvement. A similar trend emerged for the DRFi endpoint (Table S7), where TxT (MTL) showed better performance compared with other methods, achieving the best or second-best results on every task and further underlining the benefits of MTL.

Next, we evaluated the TARGET-AML dataset, which framed age and OS as multitask objectives (Table S8), and the TCGA-BRCA dataset for PAM50 subtype classification (Table S9). For TARGET-AML, TxT (MTL) exhibited better performance, attaining the best or second-best results on each metric, including a C-Index of 0.718 and lower error rates for age prediction than existing methods. Similarly, on TCGA-BRCA, TxT achieved the highest accuracy, precision, recall, and F1 scores. Overall, these results underscore the robustness of our framework, demonstrating consistent improvements in both clinical feature and survival prediction. These improvements were especially pronounced under the MTL setup, which generally led to better performance than single-task variants with particularly notable gains in survival prediction.

To further evaluate model generalizability and minimize the risk of overfitting, we also performed a more controlled internal validation using a cluster-based five-fold cross-validation strategy. Specifically, patient embeddings from the final encoder layer were clustered using k-means ($k=5$), and each cluster was used as a test fold in turn. As shown in Tables S11 and S12, TxT maintained better performance across all tasks compared to baseline models. These results confirm that TxT’s performance is not limited to random splits, but holds under more stringent and biologically structured validation schemes.

To evaluate performance beyond internal validation, we applied the model trained on the SCAN-B OS dataset to the independent METABRIC dataset [43]. As summarized in Table S13, TxT showed comparable performance relative to baseline models across clinical prediction tasks and survival estimation. Details on baseline model implementations, including preprocessing and architectural adaptations, are provided in Supplementary Methods.

### t-SNE visualization of patient embeddings

**Figure 4:**
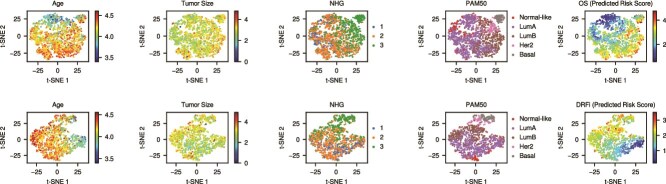
Learned latent embedding space of test samples visualized using t-SNE. Values or labels for each task are displayed as color bars or legends next to each plot. The t-SNE plots illustrate the capacity of the MTL model to capture and visually represent the relationships between various tasks in a low-dimensional embedding space. For regression and survival tasks (age, tumor size, and survival), the values were log-transformed to enhance color contrast. A separate t-SNE visualization using model prediction error as the color-coding metric is provided in Fig. S1.

To examine TxT’s learned representations, we extracted the final-layer encoder outputs for the SCAN-B OS and DRFi datasets and projected them into two dimensions using t-SNE (Fig. 4). Each column in Fig. 4 corresponds to one of the five tasks, with samples color-coded by task-related values or labels. In the OS dataset (Fig. 4, upper panel), younger (blue) and older (red) patients form distinct regions under the age task, while tumor size exhibits a weaker gradient . NHG and PAM50 subtypes cluster more clearly, with Basal samples separating from other subtypes. Notably, survival risk scores also form a gradient, indicating that TxT captures survival-related signals in its shared embedding space. A similar pattern arises for DRFi (Fig. 4, lower panel), where younger/older patients again split along different regions, and patients sharing the same NHG or PAM50 labels group together.

Overall, these t-SNE plots highlight that TxT’s shared encoder representations reflect multiple clinical and molecular factors. Although tumor size shows a less pronounced pattern, there is still evidence that samples with similar sizes tend to cluster. These observations confirm TxT’s ability to integrate both transcriptomic and clinical signals into a single embedding space, thereby facilitating the effective multitask prediction of clinical features and survival outcomes.

### Enhanced biological interpretability by clinical features

To investigate how clinical features affect gene-level attention within a shared transcriptomic context, we compared attention patterns between a multitask model and a single-task model. Genes exhibiting significant changes in attention weights upon the inclusion of clinical features were analyzed using pathway enrichment analysis to interpret the biological relevance of these changes (Fig. S2).

In the context of OS, the results revealed strong enrichment of pathways associated directly with breast cancer prognosis, including EMT and TNF-$\alpha $ signaling via NF-$\kappa $B. These pathways are critically linked to tumor progression and metastasis, particularly influencing cell adhesion, invasion, and inflammation within the tumor microenvironment [6, 44]. The enrichment of EMT-related genes underscores the ability of clinical features to guide the model in prioritizing interactions relevant to metastatic potential and tumor aggressiveness, thereby providing a clear biological basis for improved survival prediction.

Additionally, for DRFi, we observed enrichment in pathways such as EMT, ECM–receptor interaction, and Notch signaling pathway. These pathways play pivotal roles in distant metastasis by facilitating cancer cell survival in circulation, metastatic niche formation, and promoting invasive phenotypes essential for tumor dissemination [45–47]. Thus, integrating clinical features into the model notably enhances its capability to identify biologically meaningful pathways specifically associated with distant recurrence.

### Biological insights from attention-based gene interactions

**Figure 5:**
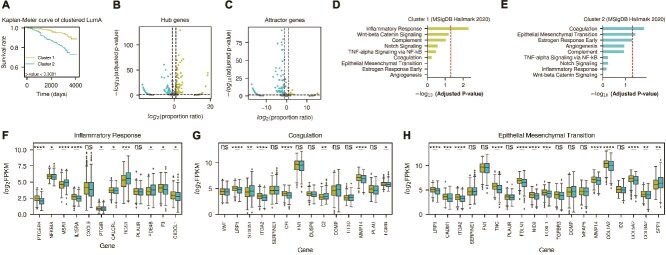
(A) Kaplan–Meier curve illustrating the survival difference between two clusters of Luminal A patients (Cluster 1 and Cluster 2). The log-rank test shows a significant survival difference. (B, C) Volcano plots showing (B) *hub genes* and (C) *attractor genes* identified from patient-specific attention-derived gene interaction networks. Genes beyond the vertical dashed lines (absolute proportion ratio $> 2$) and above the horizontal dashed line (adjusted *P*-value $<.05$) are significantly enriched in one cluster versus the other. *P*-values were computed using Fisher’s exact test and adjusted by the FDR. (D, E) Hallmark pathway enrichment analyses for (D) Cluster 1 and (E) Cluster 2, respectively. The red dashed line denotes the threshold of adjusted *P*-value $=.05$. (F–H) Box plots comparing the expression of representative genes in three hallmark processes: (F) inflammatory response, (G) coagulation, and (H) EMT. Each box represents the interquartile range, with whiskers extending 1.5 times the IQR. *P*-values were derived from *t*-tests (ns: not significant, ^*^: $<.05$, ^*^^*^: $<.01$, ^*^^*^^*^: $<.001$, ^*^^*^^*^^*^: $<.0001$).

We explored how attention-derived gene interaction networks provide biological insights into patient subgroups. Luminal A (LumA) breast cancer was selected for this analysis because it is generally less aggressive than other subtypes but exhibits significant heterogeneity in patient outcomes, making it an ideal candidate for exploring molecular differences using attention-based methods. To examine this diversity, we applied k-means clustering with $k=2$ in the latent embedding space of our multitask model for LumA patients in the test set, identifying two distinct subgroups with significantly different survival rates (Fig. 5A). Next, to obtain more detailed understanding into the molecular basis of each subgroup, we constructed attention-derived gene interaction networks for individual patients and identified hub genes and attractor genes (Fig. 5B and C). We then performed pathway enrichment analysis on these network-derived gene sets.

In the subgroup with more favorable survival, pathway analysis revealed enrichment in the inflammatory response category (Fig. 5D). Box plots (Fig. 5F) show increased expression levels of key immune-related genes such as *PTGER4*, *NFKBIA*, *IL15RA*, *PTGIR*, *CALCRL*, *F3*, and *CX3CL1*. These genes play crucial roles in various aspects of the immune response, including NF-$\kappa $B regulation, prostaglandin signaling, and T-cell activation [48]. The active immune microenvironment suggested by this gene expression profile may contribute to better tumor control and improved patient outcomes [5].

In contrast, the subgroup with worse survival exhibited enrichment of coagulation and EMT pathways (Fig. 5E). Box plots (Fig. 5G and H) show increased expression levels of genes such as *S100A1*, *C2*, and *SPP1*, which are associated with tumor progression and metastasis [4]. Interestingly, several genes typically associated with EMT and extracellular matrix remodeling are downregulated in Cluster 2, suggesting a complex interplay between pro-metastatic processes and ECM remodeling in this subgroup [49, 50].

While the initial clustering distinguished the two LumA subgroups based on latent embeddings, the subsequent construction and analysis of attention-derived gene interaction networks identified particular pathways underlying these differences in outcome. Through the multitask attention model’s capacity to capture patient-specific gene–gene relationships, we can identify immune-related connections in the better-surviving subgroup and pro-metastatic processes in the poorer-surviving subgroup.

To complement the pathway-level interpretation, we further investigated whether attention coefficients between specific gene pairs could reveal biologically meaningful patterns not captured by expression levels alone. As a case study, we focused on the interaction between *SERPINE1* and *PLAU*, two genes known to co-regulate the plasminogen activation system and associated with poor prognosis in multiple cancer types [51]. While their expression levels do not differ significantly between the two LumA subgroups (Fig. 5G), the attention coefficients for both directions are significantly elevated in the poorer-surviving subgroup (Fig. S3). This suggests that the model captures differential regulatory relevance of gene–gene interactions at the embedding level, offering insights that go beyond standard expression-based analyses. This approach provides an effective method to integrate transcriptomic data with clinical outcomes, possibly guiding refined approaches for prognostication, and targeted interventions within the LumA subtype.

### Model ablation and perturbation-based interpretation

To assess the importance of each head in the multihead self-attention layer, we performed an ablation study by selectively pruning one head at a time and evaluating the performance impact. As shown in Tables S14 and S15, removing any single head led to noticeable declines on at least one or two tasks, indicating that each head contributes unique information. In particular, pruning certain heads caused a dramatic increase in MAE for tumor size or a steep drop in C-Index. Overall, these findings confirm that multihead attention is crucial for capturing diverse task-specific patterns within a shared encoder.

We conducted ablation studies to assess the impact of PPI-based pretraining for gene embeddings. We compared two variants of TxT: (i) *Random Init*, which uses randomly initialized gene embeddings without pretraining, and (ii) *Random PPI*, which uses embeddings pretrained on a degree-preserving but biologically uninformative network generated via double-edge swaps of the STRING PPI. As shown in Tables S16 and S17, both variants showed consistent performance drops across prediction tasks. These results show that pretraining on biologically structured PPI networks provides meaningful inductive bias that improves downstream performance and interpretability.

To further investigate whether the trained model captures biologically plausible gene–risk relationships, we performed a perturbation-based simulation in which gene expression levels were systematically up- or down-regulated in the test set. We found that certain genes, when perturbed, had consistent effects on the predicted survival risk (Fig. S4). Notably, up-regulation of *CCL19* led to decreased risk scores, aligning with previous findings that higher *CCL19* expression is associated with favorable prognosis in breast cancer [52]. Similarly, down-regulation of *SERPINB5* and *FABP4* led to elevated predicted risk under the DRFi endpoint, consistent with their reported roles as prognostic markers when overexpressed [53, 54]. These results support the model’s ability to recover clinically relevant gene–outcome associations from transcriptomic inputs.

## Discussion and conclusion

In this study, we presented TxT, an MTL model that integrates transcriptomic and clinical features for survival prediction and clinical-feature inference. Using a Transformer-based architecture with multihead attention, TxT identifies intricate gene–gene interactions while simultaneously accounting for established clinical features. Our experiments showed that this combined modeling strategy can improve performance across multiple tasks compared with single-task approach. Moreover, the attention-derived gene interaction networks illuminate biological pathways associated with different patient subgroups, such as immune-related processes in the better-surviving cluster of Luminal A patients versus coagulation and EMT pathways in the poorer-surviving cluster.

Despite these promising results, certain limitations remain. Computational constraints limited our analysis to a subset of genes, potentially overlooking additional interactions and pathways. Future work could address these constraints by adopting more efficient attention mechanisms—such as local or hierarchical attention [55, 56]—or by incorporating domain knowledge to guide the model toward biologically meaningful regions of the transcriptome. Additionally, validating the generalizability of TxT in other cancers or diseases would help confirm its utility in broader clinical contexts.

Recent progress in transformer-based architectures has extended their application to single-cell RNA sequencing data, allowing more detailed analysis of cellular heterogeneity within tumors. For example, UCE [57] classifies cell roles using millions of single-cell profiles, while scGPT [58] predicts cell states and types from 33 million cells, aiding in the discovery of rare cell populations and cell lifespan prediction. Geneformer [59] integrates data from $\sim $30 million cells across numerous studies to predict the impact of gene mutations on specific diseases and support gene-based diagnostics. These single-cell transformer methods supplement bulk RNA-seq by capturing both population-level and cell-specific dynamics, providing a more thorough knowledge of tumor biology. Future iterations of TxT could potentially incorporate these single-cell approaches to further enhance its predictive capabilities and biological insights.

Overall, this study highlights the potential of an attention-based multitask approach to integrate high-dimensional transcriptomic data with clinical features, improving prediction performance while offering biologically interpretable outcomes. The comprehensive evaluation, interpretability, and biological insights derived from our model underscore its potential to advance MTL in bioinformatics and to inform broader applications in healthcare and precision medicine.

**Key Points**

- Transcriptome Transformer (TxT) effectively integrates transcriptomic data and clinical features through a multitask learning framework, significantly improving patient survival prediction.
- TxT leverages multihead attention mechanisms to dynamically model complex gene–gene interactions, offering valuable biological insights.
- Differential attention analysis demonstrates that incorporating clinical data guides the model to prioritize biologically relevant genes, highlighting pathways critical for understanding tumor progression and recurrence.

## Supplementary Material

251104_TxT_supplementary_bbaf628

## Data Availability

The SCAN-B dataset is available from Mendeley Data (https://data.mendeley.com/datasets/yzxtxn4nmd/3). The RNA sequencing-based gene expression profiles of the TCGA-BRCA and TARGET-AML datasets were downloaded from UCSC Xena (https://xenabrowser.net/datapages/) [60]. Clinical data for TCGA-BRCA were obtained from TCGA-CDR (https://gdc.cancer.gov/about-data/publications/PanCan-Clinical-2018) [61].
