# Supplementary material for: Transcriptome Transformer: improving patient survival prediction via multitask learning of transcriptomic and clinical features
Source: Brief Bioinform. 2025 Nov 25;26(6):bbaf628. doi: 10.1093/bib/bbaf628 (PMC12645844; doi:10.1093/bib/bbaf628)
Supplement: 251104_TxT_supplementary_bbaf628 [file 251104_txt_supplementary_bbaf628.pdf]

Transcriptome Transformer: Improving Patient Survival Prediction via Multi-task Learning of Transcriptomic and Clinical Features

Bonil Koo, Inyoung Sung, Sangseon Lee, and Sun Kim

Supplementary Methods

S1. N-MTLR Loss for Survival Prediction

We adopt the Neural Multi-Task Logistic Regression (N-MTLR) framework [1] for the survival prediction task. N-MTLR formulates survival modeling as a sequence of binary classification problems over a set of discretized time intervals, enabling non-parametric and censoring-aware modeling of time-to-event outcomes.

Let T denote the event time (either observed or censored), and $\delta \in \{0, 1\}$ be the event indicator, where $\delta = 1$ indicates an observed event (e.g., death), and $\delta = 0$ indicates right censoring. The time axis is divided into K discrete, non-overlapping intervals: $[0, \tau_1), [\tau_1, \tau_2), \ldots, [\tau_{K-1}, \infty)$. In our implementation, $K = 64$ intervals are used.

Let $\mathbf{z} \in \mathbb{R}^K$ denote the logits predicted by the model for a given patient, where each z_k corresponds to the log-odds of the event occurring in the k-th time interval, given survival up to that point. The logits are used to compute conditional hazard probabilities over intervals, which are then combined to estimate a discrete survival function $S(\tau_k)$.

The N-MTLR loss for an individual sample is defined as:

$$\mathcal{L}_{\text{surv}}(\mathbf{z}; T, \delta) = \begin{cases} -\log\left(\sum_{k=k_T}^{K} \frac{\exp(\sum_{j=1}^{k} z_j)}{\sum_{m=1}^{K} \exp(\sum_{j=1}^{m} z_j)}\right) & \text{if } \delta = 1 \\ -\log\left(\sum_{k=k_T+1}^{K} \frac{\exp(\sum_{j=1}^{k} z_j)}{\sum_{m=1}^{K} \exp(\sum_{j=1}^{m} z_j)}\right) & \text{if } \delta = 0 \end{cases}$$

, where k_T is the index of the time interval in which the event (or censoring) occurs. The numerator computes the cumulative probability of survival beyond time T, while the denominator normalizes across all possible survival paths.

This formulation has several desirable properties:

- It explicitly handles right-censored data.
- It makes no distributional assumptions about the underlying survival times.
- It allows the use of flexible, non-linear feature transformations.

S2. STRING Protein-Protein Interaction Network

In this study, the biological network from STRING v12.0 [2], a biological network database that represents genes as nodes and protein–protein interactions (PPIs) as edges, was used for pre-training gene embeddings. Only interactions involving proteins from Homo sapiens were included. STRING provides a confidence score (also referred to as the combined score) for PPIs, which is calculated based on literature and experimental data. This score ranges from 0 to 1000, with higher values indicating greater confidence in the interactions. To ensure a high-confidence network, only edges with a combined score exceeding 700 were included, resulting in a network comprising 16,185 nodes, 236,000 edges, and 126 connected components. Among the 126 connected components, we used the largest connected component. A connected component is defined as a maximal subset of nodes where each node is reachable from any other via a path of edges. This largest connected component consisted of 15,867 nodes and 235,783 edges, covering 98% of all nodes in the network. The remaining 125 connected components each consisted of 13 or fewer genes.

S3. Preprocessing and Sample Selection

S3.1 SCAN-B Dataset

The SCAN-B dataset [3] focuses on breast cancer patients and includes multiple replicates for certain individuals. For patients with three or more replicates, we calculated pairwise correlations among the gene expression profiles and selected the replicate exhibiting the highest average correlation. If exactly two replicates were available, we chose the sample that had been used in the original SCAN-B study. In cases where neither replicate was used in the original study, we selected the

sample with more aligned read pairs. After applying these criteria, 6,898 samples were retained for the overall survival (OS) analysis and 5,190 samples for the distant recurrence-free interval (DRFi) analysis. Additional dataset characteristics, such as the distribution of clinical features and survival times, are provided in Tables S1 and S2.

S3.2 TARGET-AML Dataset

The TARGET-AML dataset [4] comprises pediatric acute myeloid leukemia (AML) samples, where patient age is recorded in days rather than in years, and a total of 1,074 pediatric samples were included. Here, two tasks were defined: age (regression) and overall survival (OS), enabling a multi-task learning setup. Additional data characteristics, such as the distribution of age and survival times, can be found in Table S3.

S3.3 TCGA-BRCA Dataset

For the TCGA-BRCA dataset [5], we only considered primary tumor samples. In cases where multiple samples existed for the same patient, the one with a vial value "A" was chosen, following a standard convention to reduce redundancy. This selection led to a final set of 1,079 samples, each annotated with the PAM50 breast cancer subtype based on [6]. We excluded metastatic and recurrent samples to maintain a homogenous cohort of primary breast cancer cases for classification. Further descriptive statistics for subtype distribution are given in Table S4.

S3.4 Gene Filtering and Expression Normalization

All datasets underwent identical filtering and normalization procedures to ensure comparability. Genes not present in the STRING-derived network (Section S2) were excluded. Additionally, we focused on 1,000 highly variable genes selected from the MSigDB hallmark gene sets [7], ranking them by median absolute deviation (MAD) and retaining the top 1,000. Raw expression values (FPKM) were log-transformed as $\log_2(\text{FPKM} + 1)$. These log-transformed values were then scaled to the range [0, 1] using min–max values computed from the training set. The same min–max statistics were used to normalize the validation and test sets to ensure consistency during model training and evaluation.

S4. Node2vec Configuration

We employed node2vec [8] to embed each gene node from the largest connected component of the STRING network into a d_{model}-dimensional space. Following standard practices, we set:

- `walk_length` = 20
- `num_walks` = 10
- `window_size` = 10
- `p` = 1.0
- `q` = 1.0

All genes in the largest connected component were assigned continuous embeddings based on their proximity and connectivity in the network. If a gene was absent from this network or removed during the filtering steps (e.g., low expression or insufficient confidence in STRING), no embedding was generated for that gene, and it was excluded from the final model inputs.

S5. Hyperparameter Tuning

To determine the optimal number of Transformer encoder layers for the Transcriptome Transformer (TxT), we conducted hyperparameter tuning using a single random split of each dataset. We varied the number of encoder layers from 1 to 8 while keeping all other hyperparameters constant. This involved training each configuration until convergence and selecting the model that achieved the best validation performance in terms of the primary metric (F1 for classification or C-Index for survival). Early stopping was applied based on validation loss to mitigate overfitting.

Other hyperparameters were held consistent to maintain compatibility with the pre-trained node2vec embeddings and the standard Transformer architecture [9]. The model dimension (d_{model}) was set to match the dimension of the node2vec embeddings, ensuring that pre-trained gene embeddings and transcriptome-based positional encodings shared a coherent representation space. For both the SCAN-B and TARGET-AML datasets, the feed-forward layer dimension was fixed at four times d_{model}, aligning with conventional Transformer setups [9]. Detailed hyperparameter settings are provided in Table S5.

S6. Training and Evaluation Protocol

For final experiments reported in the main text, each dataset was split into training (70%), validation (10%), and test (20%) sets. This process was repeated for 10 different random seeds to ensure robust estimation of average performance. The Adam optimizer was used with a learning rate of 0.0001, and batch size was fixed at 64 for all datasets. We monitored the validation performance at the end of each epoch and performed early stopping if the validation loss did not improve for consecutive epochs. The model with the best validation performance was selected for test evaluation. Performance metrics included mean absolute error (MAE) and Spearman's correlation (SCC) for regression tasks, accuracy and F1 score for classification tasks, and concordance index (C-Index) and Integrated Brier Score (IBS) for survival prediction.

S7. Baseline Implementation Details

All baseline models were evaluated using the same train/validation/test split to ensure fair comparison. For traditional machine learning baselines (SVM, RF, and MLP), we used our own PyTorch or scikit-learn implementations, and for deep learning baselines we re-implemented each model based on their original publications, making minimal but necessary adaptations to fit our dataset.

SVM, RF, and MLP. We implemented SVM and Random Forest (RF) using `scikit-learn`, `pytorch` and `pysurvival`. For MLP, we used a 3-layer architecture ($1000 \rightarrow 128 \rightarrow 64 \rightarrow 1$) with batch normalization, LeakyReLU, and dropout. All models were trained with early stopping (patience = 10) and evaluated with standard scaling where appropriate.

OmiEmbed. We used the official OmiEmbed implementation (https://github.com/zhangxiaoyu11/OmiEmbed) and added standardization of input features as preprocessing. We enabled early stopping with patience = 10 to avoid overfitting.

T-GEM. The T-GEM model was implemented using the official open-source repository (https://github.com/TingheZhang/T-GEM) and evaluated using our unified data splits. We used the same configuration across all tasks (regression, classification, and survival).

AutoSurv. We used the official implementation of AutoSurv (https://github.com/jianglindong93/AUTOSurv). As AutoSurv relies on pre-defined pathway-level gene sets, it was not possible to use exactly the same set of genes as other baselines. To ensure comparability, we used only mRNA expression as input to both the KL-PMVAE module and the LFSurv network.

SurvConvMixer. We used the official implementation of SurvConvMixer (https://github.com/PPDPQ/SurvConvMixer). As this model also operates on pathway-defined gene sets, the input gene list differs from other models. We followed the original design by transforming gene expression into pseudo-image representations using min–max normalization.

CNN+FMAP. We used the official CNN+FMAP implementation (https://github.com/ymssll1219/Cancer-type-and-survival-prediction-based-on-transcriptomic-digital-map) and constructed a square feature map as input, following the original pre-processing and architectural design.

Supplementary Figures

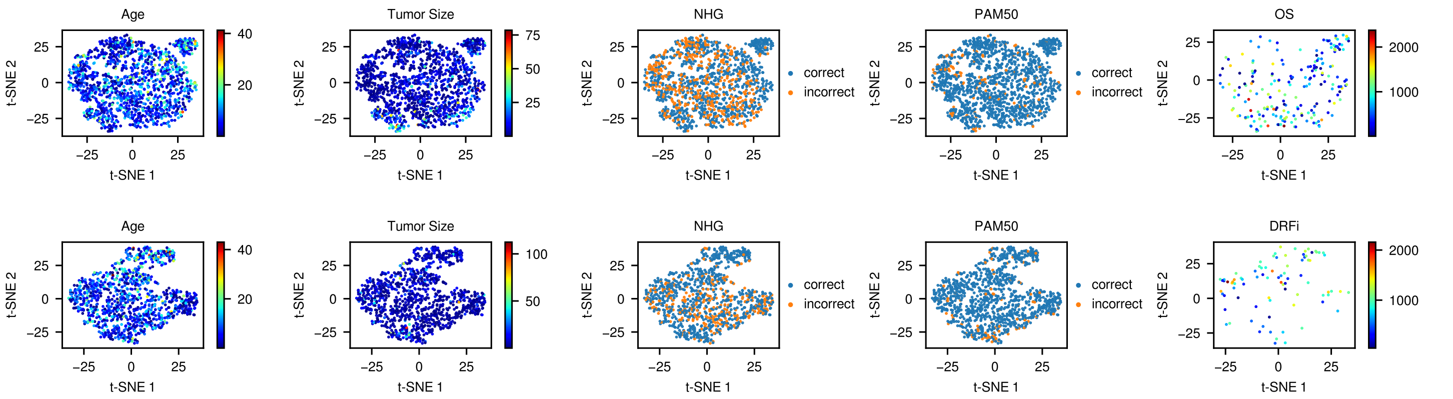

Fig. S1: t-SNE visualization of test sample embeddings, color-coded by model prediction error for each task. For regression tasks (age, tumor size), the absolute error between prediction and ground truth is used; for classification tasks (NHG, PAM50), samples are labeled as correct or incorrect; for survival tasks (OS, DRFi), the expected survival time from the predicted distribution is compared to the observed time for uncensored samples. These values are used as proxies for task-specific prediction accuracy.

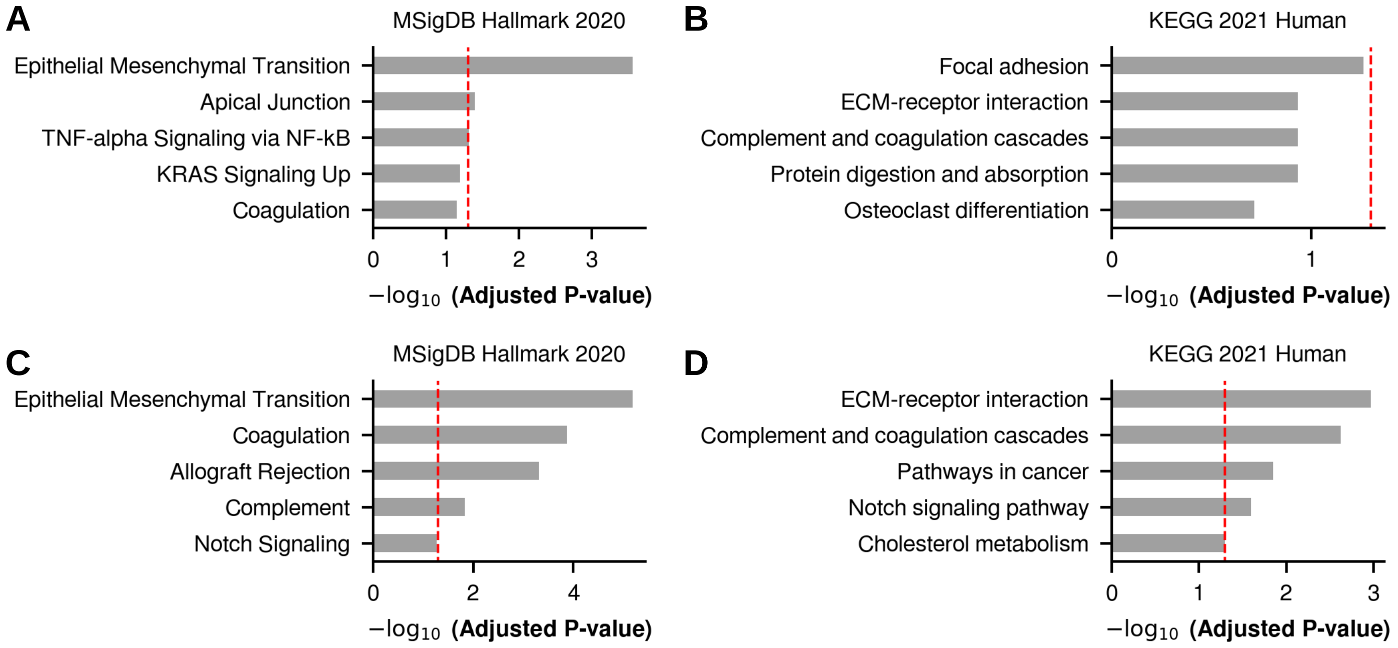

Fig. S2: Differential attention analysis was performed to identify genes whose attention increased when clinical features were included in the model. Pathway enrichment on these genes revealed biologically meaningful processes associated with breast cancer prognosis. For OS, enriched pathways included epithelial mesenchymal transition (EMT), apical junction, and TNF-α signaling via NF-κB, all linked to tumor progression and metastasis. For DRFi, pathways such as EMT, coagulation, complement, ECM-receptor interaction, and Notch signaling were enriched, implicating their roles in distant metastasis and recurrence.

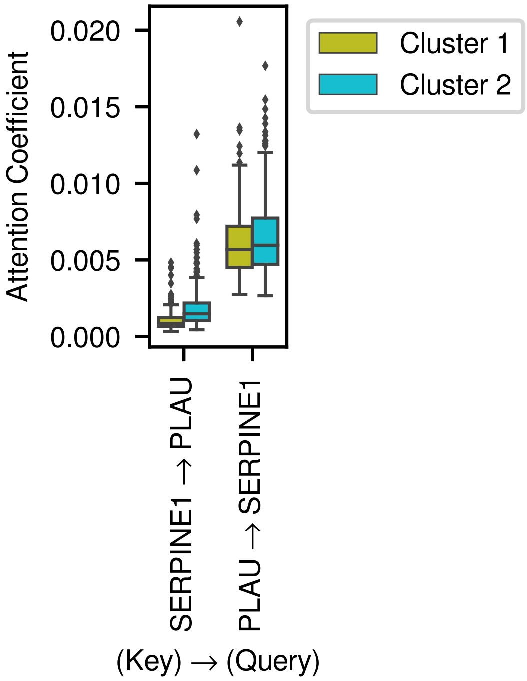

Fig. S3: Pairwise attention coefficients for the gene pair SERPINE1–PLAU across two survival-based subgroups in Luminal A patients. Each boxplot represents the attention score from the model for the indicated direction (PLAU → SERPINE1 and SERPINE1 → PLAU). Despite no significant difference in expression levels between subgroups (Fig. 5G), attention scores are significantly higher in the poor-survival group, suggesting differential functional relevance of this interaction.

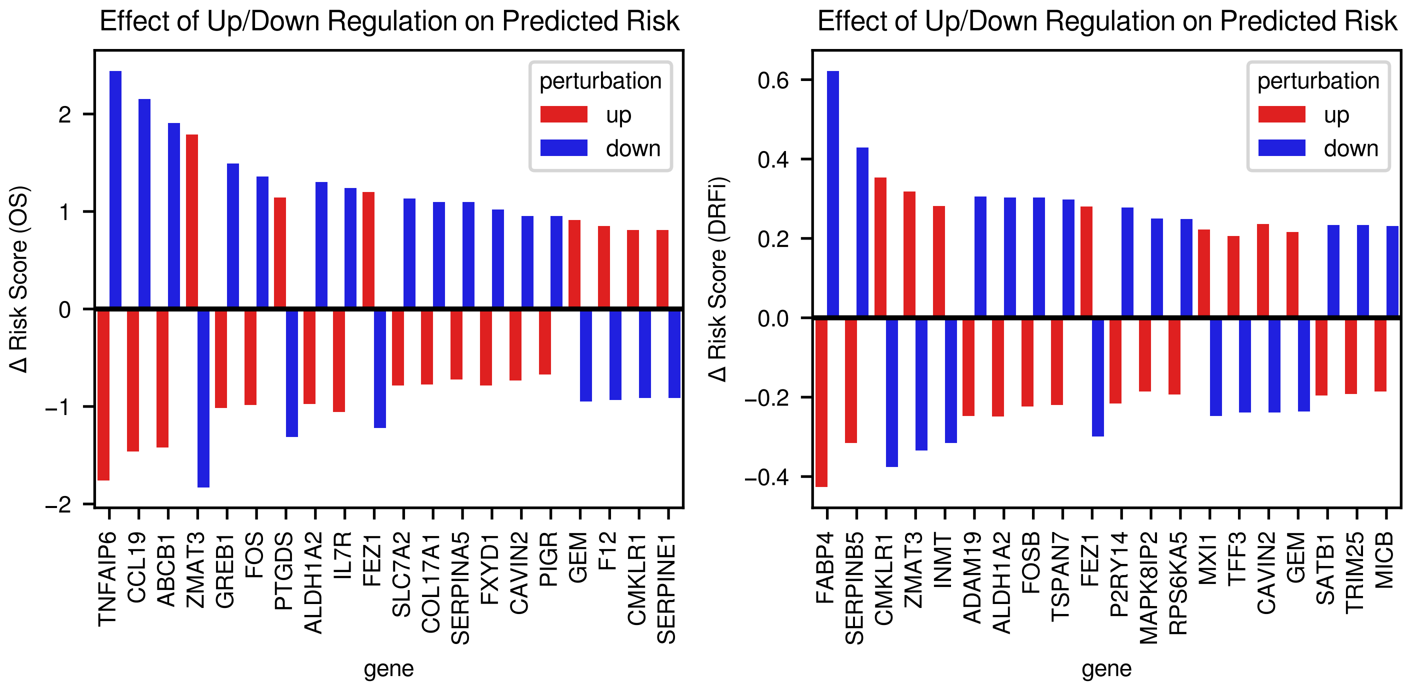

Fig. S4: Effect of *in silico* up- or down-regulation of individual genes on predicted survival risk scores. Each bar indicates the average change in predicted risk across test samples after perturbing expression of the indicated gene by ± 1 standard deviation. Left: results for OS (overall survival); Right: results for DRFi (distant recurrence-free interval). Red bars indicate up-regulation; blue bars indicate down-regulation.

Supplementary Tables

Table S1: Summary information on the SCAN-B OS dataset. For regression tasks, the provided information includes mean, standard deviation, minimum, quantiles, and maximum. For classification tasks, the number of samples among each label is presented. For survival prediction, RMST values are provided up to each year. NHG, Nottingham histological grade; OS, overall survival; RMST, restricted mean survival time.

SCAN-B OS Dataset									
Regression				Classification				Survival prediction (OS)	
Statistics	Age	statistics	Tumor size	NHG	# of samples	PAM50	# of samples	Up to (years)	RMST (days)
mean	65.5	mean	20.1	1	1130	Normal	437	2.5	898
std	13.0	std	12.3	2	3465	LumA	3584	5	1748
min	25	min	0	3	2303	LumB	1708	7.5	2538
Q1	55	Q1	12			Her2	575	10	3270
Q2	65	Q2	17			Basal	594		
Q3	75	Q3	24						
max	100	max	135						

Table S2: Summary information on the SCAN-B DRFi dataset. For regression tasks, the provided information includes mean, standard deviation, minimum, quantiles, and maximum. For classification tasks, the number of samples among each label is presented. For survival prediction, RMST values are provided up to each year. NHG, Nottingham histological grade; DRFi, distant recurrence-free interval; RMST, restricted mean survival time.

SCAN-B DRFi Dataset									
Regression				Classification				Survival prediction (DRFi)	
Statistics	Age	statistics	Tumor size	NHG	# of samples	PAM50	# of samples	Up to (years)	RMST (days)
mean	65.1	mean	20.1	1	842	Normal	318	2.5	899
std	12.9	std	12.4	2	2579	LumA	2702	5	1764
min	25	min	0	3	1769	LumB	1248	7.5	2601
Q1	55	Q1	12			Her2	454	10	3417
Q2	65	Q2	17			Basal	468		
Q3	75	Q3	24						
max	100	max	135						

Table S3: Summary information on the TARGET-AML dataset. For the age regression task, the provided information includes mean, standard deviation, minimum, quantiles, and maximum. For survival prediction, RMST values are provided up to each year. OS, overall survival; RMST, restricted mean survival time.

TARGET-AML Dataset			
Regression		Survival prediction (OS)	
Statistics	Age (days)	Up to (years)	RMST (days)
mean	3414.1	2.5	457.6
std	2323.4	5	505.7
min	10	7.5	511.6
Q1	909	10	513.1
Q2	3544		
Q3	5431.8		
max	9119		

Table S4: Summary information on the TCGA-BRCA dataset. For the PAM50 classification task, the number of samples among each label is presented.

TCGA-BRCA Dataset	
Classification	
PAM50	# of samples
Normal	40
LumA	560
LumB	207
Her2	82
Basal	190

Table S5: Hyperparameter Settings for Transcriptome Transformer (TxT)

Hyperparameter	SCAN-B Datasets	TARGET-AML Dataset	TCGA-BRCA Dataset
Feature Scaler	MinMax Scaling	MinMax Scaling	MinMax Scaling
Batch Size	64	64	64
Number of Time Intervals for Survival Prediction	64	64	-
Weight Initialization	Xavier Uniform	Xavier Uniform	Xavier Uniform
Learning Rate	0.0001	0.0001	0.0001
Early Stopping Patience	30	30	20
Number of Attention Heads	4	4	2
Dimensionality of Transformer (d_{model})	64	16	256
Dropout Rate	0.1	0.1	0.1
Dimensionality of the Feed-forward Layer	256	64	256
Number of Transformer Encoder Layers	3 for single-task learning<br>6 for multi-task learning	1	2
Aggregation Function	Flatten	Flatten	Flatten
Dimensionality of the First Hidden Layer in Task-specific Layer	128	128	128
Dimensionality of the Second Hidden Layer in Task-specific Layer	64	64	64
Slope Parameter for Leaky ReLU Activation Function	0.2	0.2	0.2

Table S6: Performance comparison on the SCAN-B OS dataset using various methods and evaluation metrics. The best performance for each metric is highlighted in bold, and the second-best is underlined. Metrics with higher values indicating better performance are marked with (↑), while those with lower values being better are marked with (↓). NHG, Nottingham histological grade; OS, overall survival; MAE, mean absolute error; RMSE, root mean squared error; PCC, Pearson's correlation coefficient; SCC, Spearman's correlation coefficient; C-Index, Concordance Index; IBS, Integrated Brier Score; SVM, support vector machine; RF, random forest; MLP, multilayer perceptron.

Method		Age				Tumor size			
		MAE(↓)	RMSE(↓)	PCC(↑)	SCC(↑)	MAE(↓)	RMSE(↓)	PCC(↑)	SCC(↑)
Single-task Learning	SVM	8.420±0.152	10.530±0.193	0.619±0.023	0.599±0.028	6.965±0.196	11.930±0.561	0.380±0.010	0.480±0.008
	RF	8.593±0.118	10.742±0.174	0.582±0.019	0.553±0.025	7.764±0.196	11.815±0.466	0.321±0.196	0.393±0.014
	MLP	8.009±0.262	10.344±0.319	0.656±0.021	0.645±0.020	7.646±0.332	11.831±0.444	0.414±0.026	0.450±0.022
	OmiEmbed	7.296±0.130	9.949±0.176	0.691±0.015	0.672±0.015	7.179±0.194	11.481±0.450	0.432±0.026	0.484±0.015
	T-GEM	15.280±5.551	18.181±5.596	0.261±0.085	0.270±0.083	11.647±4.576	16.065±3.670	0.228±0.038	0.308±0.050
	Autosurv	65.479±0.302	66.737±0.307	0.160±0.025	0.148±0.029	20.109±0.559	23.614±0.707	0.150±0.046	0.200±0.064
	SurvConvMixer	9.105±0.213	11.439±0.241	0.472±0.028	0.461±0.031	7.714±0.219	11.913±0.499	0.287±0.023	0.345±0.026
	CNN+FMAP	10.562±0.162	12.972±0.194	0.029±0.052	0.028±0.051	8.136±0.239	12.424±0.505	0.021±0.051	0.034±0.044
	TxT	7.185±0.194	9.404±0.190	0.697±0.018	**0.684±0.025**	6.778±0.171	10.535±0.418	0.442±0.025	0.485±0.007
Multi-task Learning	OmiEmbed	7.231±0.211	9.400±0.313	0.695±0.022	0.673±0.024	6.735±0.165	11.428±0.478	0.447±0.017	0.518±0.010
	TxT	**7.140±0.170**	**9.238±0.137**	**0.710±0.013**	0.681±0.021	**6.721±0.160**	**10.484±0.421**	**0.452±0.022**	**0.519±0.012**

Method		NHG				PAM50			
		Accuracy (↑)	Precision (↑)	Recall (↑)	F1 (↑)	Accuracy (↑)	Precision (↑)	Recall (↑)	F1 (↑)
Single-task Learning	SVM	0.719±0.007	**0.754±0.024**	0.609±0.009	0.623±0.012	0.905±0.005	0.895±0.009	0.843±0.014	0.865±0.010
	RF	0.698±0.008	0.723±0.016	0.581±0.010	0.585±0.012	0.882±0.008	0.891±0.014	0.781±0.014	0.821±0.014
	MLP	0.710±0.007	0.702±0.014	0.632±0.020	0.648±0.019	0.899±0.007	0.885±0.011	0.850±0.020	0.864±0.014
	OmiEmbed	0.670±0.010	0.642±0.015	0.617±0.015	0.625±0.014	0.872±0.009	0.851±0.016	0.817±0.014	0.832±0.013
	T-GEM	0.713±0.009	0.718±0.026	0.623±0.014	0.640±0.016	0.914±0.009	0.891±0.017	0.878±0.011	0.883±0.013
	Autosurv	0.632±0.045	0.505±0.073	0.510±0.043	0.482±0.046	0.664±0.057	0.486±0.161	0.494±0.126	0.464±0.135
	SurvConvMixer	0.686±0.008	0.674±0.023	0.569±0.022	0.569±0.028	0.848±0.007	0.814±0.012	0.776±0.014	0.792±0.009
	CNN+FMAP	0.617±0.020	0.592±0.031	0.557±0.020	0.564±0.020	0.817±0.011	0.794±0.013	0.746±0.011	0.766±0.011
	TxT	0.715±0.011	0.752±0.026	0.637±0.014	0.649±0.011	**0.920±0.007**	0.904±0.019	**0.892±0.017**	**0.891±0.013**
Multi-task Learning	OmiEmbed	0.712±0.008	0.719±0.017	0.618±0.011	0.635±0.014	0.898±0.005	0.882±0.009	0.846±0.020	0.860±0.013
	TxT	**0.722±0.006**	0.736±0.018	**0.675±0.016**	**0.667±0.013**	**0.920±0.007**	**0.907±0.011**	**0.892±0.018**	**0.891±0.010**

Method		OS	
		C-Index (↑)	IBS (↓)
Single-task Learning	SVM	0.645±0.012	-
	RF	0.639±0.011	0.092±0.004
	MLP	0.669±0.009	0.108±0.013
	OmiEmbed	0.674±0.080	0.098±0.006
	T-GEM	0.696±0.028	0.096±0.017
	Autosurv	0.632±0.035	0.096±0.005
	SurvConvMixer	0.672±0.010	0.100±0.002
	CNN+FMAP	0.677±0.020	0.090±0.029
	TxT	0.701±0.014	0.087±0.004
Multi-task Learning	OmiEmbed	0.778±0.028	0.112±0.019
	TxT	**0.797±0.017**	**0.084±0.007**

Table S7: Performance comparison on the SCAN-B DRFi dataset using various methods and evaluation metrics. The best performance for each metric is highlighted in bold, and the second-best is underlined. Metrics with higher values indicating better performance are marked with (↑), while those with lower values being better are marked with (↓). NHG, Nottingham histological grade; DRFi, distant recurrence-free interval; MAE, mean absolute error; RMSE, root mean squared error; PCC, Pearson's correlation coefficient; SCC, Spearman's correlation coefficient; C-Index, Concordance Index; IBS, Integrated Brier Score; SVM, support vector machine; RF, random forest; MLP, multilayer perceptron.

Method		Age				Tumor size			
		MAE(↓)	RMSE(↓)	PCC(↑)	SCC(↑)	MAE(↓)	RMSE(↓)	PCC(↑)	SCC(↑)
Single-task Learning	SVM	8.525±0.178	10.678±0.204	0.601±0.013	0.588±0.016	7.041±0.256	11.912±0.756	0.371±0.023	0.472±0.021
	RF	8.587±0.182	10.775±0.171	0.576±0.019	0.557±0.019	7.836±0.289	11.822±0.647	0.306±0.026	0.379±0.024
	MLP	8.138±0.258	10.490±0.331	0.641±0.022	0.641±0.024	7.732±0.216	11.805±0.589	0.422±0.020	0.454±0.019
	OmiEmbed	7.394±0.146	9.564±0.239	0.682±0.018	0.668±0.021	7.219±0.229	11.459±0.652	0.428±0.023	0.481±0.026
	T-GEM	18.323±4.538	21.408±4.424	0.181±0.125	0.195±0.120	11.811±3.408	16.018±2.989	0.227±0.050	0.309±0.042
	Autosurv	65.299±0.441	66.552±0.437	0.116±0.105	0.109±0.101	20.074±0.288	23.556±0.552	0.146±0.063	0.199±0.103
	SurvConvMixer	9.227±0.217	11.532±0.257	0.454±0.023	0.451±0.021	7.856±0.340	11.943±0.705	0.261±0.017	0.317±0.018
	CNN+FMAP	10.557±0.269	12.944±0.261	0.010±0.025	0.013±0.021	8.189±0.181	12.355±0.735	0.056±0.068	0.071±0.074
	TxT	7.349±0.128	9.522±0.208	0.711±0.020	0.679±0.015	6.920±0.221	10.801±0.630	0.399±0.020	0.439±0.029
Multi-task Learning	OmiEmbed	7.351±0.121	9.528±0.155	0.683±0.015	0.669±0.016	6.786±0.295	11.340±0.823	0.448±0.033	0.517±0.028
	TxT	**7.328±0.121**	**9.410±0.248**	**0.720±0.017**	**0.684±0.017**	**6.717±0.216**	**10.268±0.743**	**0.451±0.023**	**0.521±0.022**

Method		NHG				PAM50			
		Accuracy (↑)	Precision (↑)	Recall (↑)	F1 (↑)	Accuracy (↑)	Precision (↑)	Recall (↑)	F1 (↑)
Single-task Learning	SVM	0.726±0.011	**0.757±0.022**	0.617±0.011	0.633±0.015	0.906±0.006	0.903±0.009	0.849±0.013	0.872±0.009
	RF	0.707±0.011	0.720±0.029	0.589±0.012	0.594±0.018	0.882±0.008	0.899±0.011	0.785±0.012	0.825±0.011
	MLP	0.717±0.013	0.713±0.023	0.628±0.028	0.643±0.028	0.898±0.006	0.885±0.010	0.852±0.016	0.866±0.010
	OmiEmbed	0.675±0.014	0.644±0.018	0.627±0.017	0.633±0.016	0.869±0.009	0.852±0.011	0.819±0.017	0.833±0.013
	T-GEM	0.721±0.006	0.728±0.021	0.624±0.012	0.643±0.013	0.912±0.006	0.899±0.019	0.875±0.017	0.884±0.012
	Autosurv	0.600±0.069	0.530±0.054	0.529±0.039	0.500±0.037	0.639±0.168	0.544±0.123	0.509±0.113	0.477±0.136
	SurvConvMixer	0.685±0.010	0.668±0.027	0.576±0.013	0.580±0.021	0.845±0.006	0.812±0.013	0.776±0.017	0.791±0.013
	CNN+FMAP	0.616±0.023	0.580±0.025	0.557±0.018	0.560±0.013	0.800±0.015	0.769±0.027	0.730±0.017	0.744±0.013
	TxT	0.720±0.010	0.741±0.022	0.638±0.021	0.661±0.011	**0.923±0.011**	**0.915±0.012**	0.901±0.017	0.903±0.012
Multi-task Learning	OmiEmbed	0.715±0.012	0.704±0.022	0.633±0.016	0.648±0.018	0.898±0.007	0.887±0.015	0.854±0.015	0.867±0.011
	TxT	**0.735±0.019**	0.743±0.021	**0.657±0.013**	**0.667±0.020**	0.922±0.012	0.910±0.009	**0.904±0.014**	**0.907±0.017**

Method		DRFi	
		C-Index (↑)	IBS (↓)
Single-task Learning	SVM	0.608±0.039	-
	RF	0.657±0.056	0.061±0.005
	MLP	0.693±0.024	0.070±0.006
	OmiEmbed	0.704±0.095	0.079±0.015
	T-GEM	0.716±0.065	0.131±0.008
	Autosurv	0.713±0.040	0.062±0.005
	SurvConvMixer	0.678±0.026	0.062±0.001
	CNN+FMAP	0.609±0.056	0.066±0.006
	TxT	0.718±0.062	0.059±0.004
Multi-task Learning	OmiEmbed	0.763±0.119	0.064±0.006
	TxT	**0.787±0.062**	**0.055±0.006**

Table S8: Performance comparison on the TARGET-AML dataset using various methods and evaluation metrics. The best performance for each metric is highlighted in bold, and the second-best is underlined. Metrics with higher values indicating better performance are marked with (↑), while those with lower values being better are marked with (↓). OS, overall survival; MAE, mean absolute error; RMSE, root mean squared error; PCC, Pearson's correlation coefficient; SCC, Spearman's correlation coefficient; C-Index, Concordance Index; IBS, Integrated Brier Score; SVM, support vector machine; RF, random forest; MLP, multilayer perceptron.

Method		Age (days)				OS	
		MAE (↓)	RMSE (↓)	PCC (↑)	SCC (↑)	C-Index (↑)	IBS (↓)
Single-task Learning	SVM	1981.906±253.434	2187.612±211.764	0.525±0.126	0.594±0.103	0.657±0.043	-
	RF	1609.436±228.695	1775.487±186.858	0.584±0.102	0.643±0.073	0.609±0.030	0.188±0.014
	MLP	1415.211±184.889	1118.825±156.947	0.681±0.032	0.689±0.031	0.665±0.028	0.077±0.015
	OmiEmbed	1388.642±198.986	1015.540±173.564	0.695±0.068	0.693±0.075	0.673±0.029	0.079±0.016
	T-GEM	1872.811±113.755	2259.856±122.264	0.305±0.158	0.346±0.168	0.676±0.021	0.073±0.015
	SurvConvMixer	1955.102±524.390	2466.293±608.884	0.594±0.044	0.592±0.043	0.523±0.012	0.066±0.002
	CNN+FMAP	2078.942±62.393	2339.453±58.566	0.027±0.097	0.036±0.093	0.508±0.020	0.084±0.021
	TxT	1298.357±104.543	**1005.021±155.064**	0.705±0.065	0.709±0.079	0.708±0.021	0.071±0.019
Multi-task Learning	OmiEmbed	1366.107±304.190	1086.324±244.428	0.718±0.139	0.721±0.138	0.700±0.024	0.070±0.023
	TxT	**1204.307±198.251**	1011.892±167.626	**0.721±0.069**	**0.740±0.073**	**0.718±0.031**	**0.069±0.039**

Table S9: Performance comparison on the TCGA-BRCA dataset using various methods and evaluation metrics. The best performance for each metric is highlighted in bold, and the second-best is underlined. SVM, support vector machine; RF, random forest; MLP, multilayer perceptron.

Method	Accuracy	Precision	Recall	F1
SVM	0.869 ± 0.022	0.840 ± 0.092	0.722 ± 0.041	0.743 ± 0.050
RF	0.850 ± 0.024	0.827 ± 0.097	0.666 ± 0.049	0.695 ± 0.059
MLP	0.870 ± 0.029	0.820 ± 0.060	0.788 ± 0.056	0.789 ± 0.056
OmiEmbed	0.865 ± 0.023	0.840 ± 0.067	0.736 ± 0.061	0.755 ± 0.065
T-GEM	0.875 ± 0.021	0.817 ± 0.051	0.812 ± 0.056	0.807 ± 0.049
SurvConvMixer	0.857 ± 0.020	0.787 ± 0.070	0.742 ± 0.040	0.749 ± 0.045
CNN+FMAP	0.691 ± 0.026	0.585 ± 0.061	0.515 ± 0.026	0.522 ± 0.027
TxT	**0.894 ± 0.026**	**0.862 ± 0.048**	**0.817 ± 0.058**	**0.827 ± 0.052**

Table S10: Paired t-test results comparing TxT and OmiEmbed on survival prediction tasks across multiple datasets and learning setups, based on C-Index scores. TxT consistently outperforms the baseline with statistical significance in both single-task (STL) and multi-task (MTL) learning settings. Asterisks denote significance levels (-: not significant, *: $p < 0.05$, **: $p < 0.01$, ***: $p < 0.001$).

Dataset	Learning	Baseline	TxT Mean	Baseline Mean	p-value	Significance
SCAN-B OS	STL	OmiEmbed	0.708	0.673	0.0019	**
	MTL		0.797	0.764	0.0023	**
SCAN-B DRFi	STL		0.718	0.674	0.00044	***
	MTL		0787	0.763	0.020	*
TAGET-AML	STL		0.708	0.673	0.016	*
	MTL		0.718	0.700	0.164	-

Table S11: Performance comparison under 5-fold cross-validation using cluster-based splits on SCAN-B OS dataset. The best performance for each metric is highlighted in bold, and the second-best is underlined. Metrics with higher values indicating better performance are marked with (↑), while those with lower values being better are marked with (↓). NHG, Nottingham histological grade; OS, overall survival; MAE, mean absolute error; RMSE, root mean squared error; PCC, Pearson's correlation coefficient; SCC, Spearman's correlation coefficient; C-Index, Concordance Index; IBS, Integrated Brier Score; SVM, support vector machine; RF, random forest; MLP, multilayer perceptron.

Method		Age				Tumor size			
		MAE(↓)	RMSE(↓)	PCC(↑)	SCC(↑)	MAE(↓)	RMSE(↓)	PCC(↑)	SCC(↑)
Single-task Learning	SVM	11.013±0.735	8.842±0.562	0.553±0.055	0.526±0.083	12.166±1.037	7.590±0.735	0.286±0.085	0.338±0.076
	RF	12.895±1.763	10.715±1.686	0.223±0.221	0.196±0.178	12.033±1.106	7.891±0.539	0.231±0.125	0.301±0.117
	MLP	27.645±10.769	24.955±11.455	0.630±0.052	0.617±0.066	14.167±0.857	10.330±1.137	0.396±0.039	0.440±0.037
	OmiEmbed	10.598±1.733	8.356±1.556	0.546±0.225	0.535±0.213	11.926±0.680	7.881±0.399	0.276±0.033	0.365±0.017
	T-GEM	26.407±4.674	23.351±4.605	0.105±0.121	0.120±0.121	16.164±2.712	11.909±3.014	0.199±0.044	0.280±0.060
	Autosurv	66.331±2.428	65.062±2.556	0.123±0.018	0.105±0.030	23.567±1.404	20.126±1.173	0.138±0.032	0.187±0.054
	SurvConvMixer	11.576±0.735	9.253±0.572	0.449±0.051	0.443±0.052	11.869±0.773	7.824±0.615	0.266±0.035	0.314±0.032
	CNN+FMAP	13.200±0.966	10.854±0.941	0.011±0.046	0.009±0.042	12.416±0.925	8.231±0.307	0.036±0.042	0.039±0.061
	TxT	**9.543±0.532**	**7.390±0.451**	**0.682±0.029**	**0.661±0.052**	**11.437±0.722**	**6.863±0.513**	**0.431±0.011**	**0.498±0.018**
Multi-task Learning	OmiEmbed	11.000±0.775	8.875±0.610	0.587±0.049	0.570±0.072	11.945±0.904	7.092±0.556	0.358±0.017	0.453±0.014
	TxT	9.783±0.508	7.574±0.412	0.673±0.020	0.657±0.042	11.487±0.744	7.267±0.509	0.409±0.037	0.450±0.035

Method		NHG				PAM50			
		Accuracy (↑)	Precision (↑)	Recall (↑)	F1 (↑)	Accuracy (↑)	Precision (↑)	Recall (↑)	F1 (↑)
Single-task Learning	SVM	0.664±0.031	0.627±0.019	0.594±0.043	0.604±0.037	0.897±0.011	0.873±0.019	0.843±0.046	0.853±0.024
	RF	0.647±0.105	0.578±0.237	0.563±0.133	0.549±0.190	0.766±0.226	0.677±0.339	0.653±0.276	0.645±0.314
	MLP	0.704±0.016	0.690±0.025	0.603±0.018	0.618±0.018	0.866±0.027	0.849±0.030	0.810±0.040	0.826±0.035
	OmiEmbed	0.704±0.027	0.689±0.030	0.610±0.028	0.618±0.034	0.894±0.015	0.870±0.026	0.840±0.013	0.852±0.018
	T-GEM	**0.719±0.019**	**0.753±0.021**	0.598±0.014	0.611±0.011	0.894±0.013	0.874±0.018	0.839±0.013	0.854±0.012
	Autosurv	0.571±0.035	0.511±0.026	0.476±0.050	0.454±0.073	0.607±0.125	0.430±0.222	0.401±0.136	0.358±0.158
	SurvConvMixer	0.687±0.022	0.665±0.031	0.561±0.016	0.557±0.021	0.849±0.015	0.818±0.021	0.776±0.039	0.791±0.030
	CNN+FMAP	0.603±0.047	0.565±0.024	0.557±0.015	0.552±0.013	0.801±0.026	0.758±0.041	0.747±0.045	0.748±0.035
	TxT	**0.719±0.021**	0.715±0.022	**0.621±0.019**	**0.639±0.019**	**0.912±0.008**	0.892±0.024	**0.862±0.007**	**0.874±0.011**
Multi-task Learning	OmiEmbed	0.702±0.027	0.712±0.031	0.579±0.026	0.586±0.027	0.881±0.012	**0.893±0.018**	0.774±0.024	0.816±0.019
	TxT	0.710±0.024	0.694±0.020	0.620±0.022	0.638±0.023	0.904±0.011	0.890±0.022	0.838±0.018	0.861±0.018

Method		OS	
		C-Index (↑)	IBS (↓)
Single-task Learning	SVM	0.635±0.037	-
	RF	0.606±0.026	0.102±0.017
	MLP	0.617±0.024	0.130±0.021
	OmiEmbed	0.638±0.108	0.104±0.019
	T-GEM	0.687±0.018	0.105±0.016
	Autosurv	0.632±0.035	0.104±0.014
	SurvConvMixer	0.639±0.032	0.106±0.013
	CNN+FMAP	0.577±0.034	0.107±0.014
	TxT	0.688±0.019	0.103±0.013
Multi-task Learning	OmiEmbed	0.749±0.059	0.105±0.018
	TxT	**0.752±0.040**	**0.095±0.014**

Table S12: Performance comparison under 5-fold cross-validation using cluster-based splits on SCAN-B DRFi dataset. The best performance for each metric is highlighted in bold, and the second-best is underlined. Metrics with higher values indicating better performance are marked with (↑), while those with lower values being better are marked with (↓). NHG, Nottingham histological grade; DRFi, distant recurrence-free interval; MAE, mean absolute error; RMSE, root mean squared error; PCC, Pearson's correlation coefficient; SCC, Spearman's correlation coefficient; C-Index, Concordance Index; IBS, Integrated Brier Score; SVM, support vector machine; RF, random forest; MLP, multilayer perceptron.

Method		Age				Tumor size			
		MAE(↓)	RMSE(↓)	PCC(↑)	SCC(↑)	MAE(↓)	RMSE(↓)	PCC(↑)	SCC(↑)
Sigle-task Learning	SVM	12.216±1.720	9.961±1.662	0.409±0.243	0.409±0.251	12.693±0.692	8.283±1.319	0.221±0.145	0.273±0.139
	RF	11.214±1.380	8.953±1.362	0.473±0.243	0.460±0.255	12.186±0.615	7.200±0.325	0.353±0.017	0.454±0.031
	MLP	34.100±13.545	31.495±14.474	0.621±0.045	0.607±0.051	17.134±1.786	13.350±1.778	0.413±0.037	0.428±0.049
	OmiEmbed	11.140±0.584	9.007±0.583	0.573±0.057	0.556±0.072	12.039±0.647	7.972±0.389	0.292±0.032	0.369±0.028
	T-GEM	23.742±5.490	20.725±5.342	0.101±0.056	0.125±0.050	16.334±4.462	11.679±4.678	0.219±0.028	0.304±0.051
	Autosurv	65.793±2.969	64.549±3.094	0.164±0.034	0.146±0.034	23.813±0.961	20.276±0.939	0.099±0.047	0.164±0.076
	SurvConvMixer	11.512±0.504	9.190±0.428	0.439±0.071	0.434±0.078	12.153±0.772	8.021±0.406	0.246±0.046	0.300±0.028
	CNN+FMAP	13.307±0.785	10.939±0.864	-0.008±0.029	-0.007±0.027	12.525±0.672	8.379±0.271	0.052±0.063	0.076±0.079
	TxT	9.900±0.400	7.684±0.317	0.649±0.037	0.631±0.058	**11.683±0.619**	**6.996±0.338**	**0.423±0.009**	**0.494±0.008**
Multi-task Learning	OmiEmbed	11.128±0.555	8.962±0.510	0.538±0.069	0.511±0.084	12.117±0.898	8.006±0.882	0.254±0.138	0.339±0.134
	TxT	**9.824±0.319**	**7.583±0.263**	**0.656±0.036**	**0.639±0.056**	11.751±0.715	7.469±0.358	0.408±0.028	0.443±0.017

Method		NHG				PAM50			
		Accuracy (↑)	Precision (↑)	Recall (↑)	F1 (↑)	Accuracy (↑)	Precision (↑)	Recall (↑)	F1 (↑)
Single-task Learning	SVM	0.708±0.080	0.708±0.229	0.578±0.132	0.589±0.177	0.895±0.016	0.883±0.025	0.827±0.011	0.849±0.012
	RF	0.702±0.029	0.703±0.035	0.577±0.016	0.580±0.013	0.705±0.132	0.570±0.319	0.524±0.259	0.501±0.288
	MLP	0.716±0.031	0.721±0.048	0.605±0.015	0.622±0.010	0.880±0.016	0.892±0.012	0.768±0.027	0.810±0.023
	OmiEmbed	0.668±0.033	0.622±0.018	0.601±0.019	0.609±0.017	0.861±0.022	0.840±0.030	0.806±0.017	0.819±0.013
	T-GEM	**0.720±0.031**	**0.752±0.014**	0.595±0.018	0.604±0.025	0.895±0.015	0.883±0.026	0.840±0.018	0.855±0.015
	Autosurv	0.590±0.069	0.484±0.044	0.481±0.031	0.446±0.056	0.666±0.071	0.569±0.061	0.572±0.064	0.533±0.052
	SurvConvMixer	0.685±0.035	0.649±0.029	0.563±0.021	0.567±0.020	0.850±0.020	0.816±0.022	0.777±0.034	0.792±0.030
	CNN+FMAP	0.616±0.037	0.567±0.030	0.541±0.021	0.540±0.023	0.780±0.012	0.755±0.013	0.723±0.027	0.732±0.014
	TxT	0.711±0.029	0.687±0.029	**0.618±0.016**	**0.632±0.014**	**0.915±0.013**	0.891±0.023	**0.868±0.016**	**0.877±0.008**
Multi-task Learning	OmiEmbed	0.707±0.027	0.708±0.025	0.588±0.021	0.596±0.019	0.860±0.079	0.783±0.182	0.746±0.196	0.750±0.209
	TxT	0.714±0.031	0.699±0.016	0.612±0.028	0.626±0.029	0.905±0.008	**0.898±0.007**	0.839±0.008	0.864±0.006

Method		DRFi	
		C-Index (↑)	IBS (↓)
Single-task Learning	SVM	0.614±0.040	-
	RF	0.628±0.081	0.068±0.011
	MLP	0.675±0.035	**0.064±0.012**
	OmiEmbed	0.640±0.118	0.067±0.012
	T-GEM	0.685±0.046	0.096±0.023
	Autosurv	0.690±0.027	0.068±0.011
	SurvConvMixer	0.662±0.042	0.071±0.013
	CNN+FMAP	0.576±0.037	0.077±0.020
	TxT	0.699±0.065	0.067±0.012
Multi-task Learning	OmiEmbed	0.707±0.051	0.076±0.015
	TxT	**0.759±0.065**	0.074±0.023

Table S13: Performance of models trained on SCAN-B OS dataset and externally validated on the METABRIC dataset. The best performance for each metric is highlighted in bold, and the second-best is underlined. Metrics with higher values indicating better performance are marked with (↑), while those with lower values being better are marked with (↓). NHG, Nottingham histological grade; OS, overall survival; MAE, mean absolute error; RMSE, root mean squared error; PCC, Pearson's correlation coefficient; SCC, Spearman's correlation coefficient; C-Index, Concordance Index; IBS, Integrated Brier Score; SVM, support vector machine; RF, random forest; MLP, multilayer perceptron.

Method		Age				Tumor size			
		MAE(↓)	RMSE(↓)	PCC(↑)	SCC(↑)	MAE(↓)	RMSE(↓)	PCC(↑)	SCC(↑)
Single-task Learning	SVM	13.322±0.794	10.907±0.606	0.098±0.244	0.093±0.255	17.746±0.258	11.339±0.279	**0.270±0.011**	**0.340±0.011**
	RF	14.756±1.020	11.734±0.917	0.389±0.117	0.395±0.111	17.613±0.251	11.488±0.227	0.187±0.020	0.232±0.016
	MLP	17.319±4.200	14.267±3.994	0.236±0.189	0.234±0.182	18.875±3.601	13.282±4.168	0.166±0.151	0.193±0.196
	OmiEmbed	12.923±0.081	10.352±0.060	**0.590±0.005**	**0.589±0.005**	18.218±1.049	11.466±1.127	0.122±0.032	0.166±0.042
	T-GEM	16.142±4.369	13.289±4.194	0.228±0.271	0.232±0.269	23.654±13.343	19.195±14.888	0.088±0.162	0.096±0.198
	TxT	**11.338±0.257**	**8.756±0.189**	0.559±0.017	0.548±0.016	16.816±0.095	**10.020±0.089**	0.254±0.005	0.317±0.005
Multi-task Learning	OmiEmbed	12.935±0.125	10.378±0.078	0.484±0.013	0.479±0.013	18.118±1.526	12.927±1.741	0.149±0.022	0.167±0.025
	TxT	12.199±0.517	9.419±0.392	0.521±0.038	0.508±0.038	**15.785±0.188**	10.972±0.380	0.110±0.011	0.131±0.021

Method		NHG				PAM50			
		Accuracy (↑)	Precision (↑)	Recall (↑)	F1 (↑)	Accuracy (↑)	Precision (↑)	Recall (↑)	F1 (↑)
Single-task Learning	SVM	0.673±0.005	**0.660±0.025**	0.512±0.005	0.498±0.009	0.745±0.021	0.779±0.015	0.669±0.017	0.684±0.018
	RF	0.653±0.008	0.561±0.020	0.498±0.004	0.488±0.008	0.724±0.008	0.776±0.009	0.610±0.009	0.636±0.010
	MLP	0.655±0.011	0.601±0.019	0.519±0.014	0.508±0.016	0.712±0.012	0.733±0.017	0.624±0.012	0.656±0.012
	OmiEmbed	0.638±0.055	0.567±0.030	0.541±0.027	0.529±0.039	0.754±0.006	**0.817±0.013**	0.622±0.008	0.641±0.009
	T-GEM	0.640±0.015	0.601±0.014	0.560±0.011	0.559±0.010	0.771±0.006	0.809±0.004	0.669±0.006	0.687±0.008
	TxT	**0.691±0.002**	0.635±0.027	0.568±0.026	0.566±0.037	0.762±0.029	0.788±0.031	0.674±0.011	0.697±0.012
Multi-task Learning	OmiEmbed	0.558±0.018	0.510±0.014	0.515±0.019	0.495±0.019	0.737±0.009	0.787±0.010	0.622±0.010	0.655±0.010
	TxT	0.656±0.014	0.588±0.014	**0.581±0.016**	**0.575±0.008**	**0.792±0.005**	0.812±0.005	**0.698±0.009**	**0.722±0.010**

Method		OS	
		C-Index (↑)	IBS (↓)
Single-task Learning	SVM	0.512±0.083	-
	RF	0.538±0.061	0.473±0.012
	MLP	0.561±0.030	0.352±0.056
	OmiEmbed	0.578±0.010	0.457±0.000
	T-GEM	0.561±0.047	0.347±0.040
	TxT	0.613±0.011	0.074±0.029
Multi-task Learning	OmiEmbed	0.615±0.012	0.578±0.001
	TxT	**0.619±0.019**	**0.071±0.086**

Table S14: Ablation study results for pruning individual heads of the first layer in the multi-task learning model on the SCAN-B OS dataset. Cases with the most significant performance degradation upon pruning each head are highlighted in bold. Metrics where higher values indicate better performance are marked with (↑), while lower values are better for metrics marked with (↓). NHG, Nottingham histological grade; OS, overall survival; MAE, mean absolute error; SCC, Spearman's correlation coefficient; ACC, accuracy; C-Index, Concordance Index; IBS, Integrated Brier Score; MHSA, multi-head self-attention; GWFF, gene-wise feed-forward.

Pruned layer/head	Age		Tumor Size		NHG		PAM50		OS	
	MAE(↓)	SCC(↑)	MAE(↓)	SCC(↑)	ACC(↑)	F1(↑)	ACC(↑)	F1(↑)	C-Index(↑)	IBS(↓)
Original	7.170	0.681	6.788	0.509	0.722	0.665	0.928	0.891	0.790	0.081
Layer 1	10.910	0.049	158.971	-0.233	0.325	0.164	0.098	0.081	0.489	0.729
Layer 2	8.457	0.666	29.640	0.372	0.587	0.432	0.771	0.705	0.677	0.111
Layer 3	7.238	0.677	9.691	0.488	0.675	0.534	0.678	0.665	0.733	0.104
Layer 4	7.321	0.676	7.817	0.500	0.698	0.620	0.867	0.831	0.728	0.102
Layer 5	7.204	0.681	9.206	0.503	0.673	0.535	0.802	0.763	0.734	0.101
Layer 6	7.544	0.678	9.325	0.494	0.681	0.547	0.878	0.838	0.736	0.102
MHSA (Layer 1)	15.261	0.013	24.379	-0.249	0.325	0.164	0.470	0.261	0.433	0.175
MHSA (Layer 2)	9.353	0.448	11.557	0.419	0.529	0.385	0.470	0.509	0.665	0.108
MHSA (Layer 3)	7.371	0.676	7.265	0.483	0.697	0.627	0.856	0.821	0.721	0.104
MHSA (Layer 4)	7.331	0.675	7.364	0.499	0.702	0.635	0.880	0.836	0.723	0.103
MHSA (Layer 5)	7.135	0.683	7.856	0.514	0.679	0.566	0.860	0.828	0.731	0.101
MHSA (Layer 6)	7.530	0.680	8.815	0.501	0.678	0.541	0.891	0.868	0.733	0.103
GWFF (Layer 1)	19.494	0.437	179.658	0.390	0.650	0.478	0.150	0.147	0.694	0.658
GWFF (Layer 2)	8.237	0.670	13.220	0.421	0.676	0.574	0.833	0.716	0.710	0.111
GWFF (Layer 3)	7.281	0.679	9.486	0.481	0.687	0.590	0.852	0.813	0.737	0.101
GWFF (Layer 4)	7.283	0.679	8.777	0.502	0.684	0.590	0.870	0.821	0.735	0.102
GWFF (Layer 5)	7.239	0.678	9.347	0.499	0.692	0.583	0.878	0.844	0.734	0.102
GWFF (Layer 6)	7.192	0.678	8.492	0.500	0.701	0.620	0.888	0.855	0.731	0.101
Head 1 (Layer 1)	**11.127**	**0.621**	7.647	0.470	0.676	0.536	0.761	**0.599**	0.733	**0.103**
Head 2 (Layer 1)	8.195	0.636	**25.149**	**0.375**	0.678	**0.500**	0.782	0.682	0.735	0.099
Head 3 (Layer 1)	7.228	0.650	6.955	0.481	**0.672**	0.539	**0.700**	0.662	0.731	0.096
Head 4 (Layer 1)	8.499	0.640	7.560	0.500	0.679	0.554	0.844	0.732	**0.725**	0.098
Head 1 (Layer 2)	7.322	0.666	7.117	0.519	0.661	0.536	0.753	0.737	0.729	0.102
Head 2 (Layer 2)	7.217	0.675	7.168	0.522	0.704	0.622	0.886	0.847	0.729	0.100
Head 3 (Layer 2)	7.244	0.677	10.639	0.444	0.674	0.546	0.888	0.863	0.723	0.104
Head 4 (Layer 2)	8.149	0.659	10.012	0.494	0.684	0.596	0.860	0.756	0.721	0.101
Head 1 (Layer 3)	7.187	0.68	7.680	0.517	0.699	0.621	0.885	0.856	0.729	0.102
Head 2 (Layer 3)	7.169	0.681	7.332	0.512	0.695	0.609	0.885	0.849	0.724	0.102
Head 3 (Layer 3)	7.191	0.68	8.288	0.516	0.701	0.609	0.883	0.853	0.730	0.102
Head 4 (Layer 3)	7.418	0.675	7.913	0.513	0.699	0.620	0.886	0.839	0.727	0.102
Head 1 (Layer 4)	7.141	0.681	8.030	0.508	0.697	0.586	0.900	0.866	0.727	0.101
Head 2 (Layer 4)	7.177	0.679	8.001	0.505	0.693	0.629	0.873	0.826	0.727	0.102
Head 3 (Layer 4)	7.185	0.678	7.701	0.506	0.698	0.625	0.879	0.831	0.729	0.102
Head 4 (Layer 4)	7.168	0.681	7.768	0.513	0.699	0.609	0.899	0.870	0.729	0.101
Head 1 (Layer 5)	7.154	0.68	8.131	0.507	0.701	0.619	0.884	0.854	0.731	0.101
Head 2 (Layer 5)	7.145	0.68	8.114	0.511	0.693	0.591	0.904	0.876	0.731	0.101
Head 3 (Layer 5)	7.145	0.682	7.764	0.516	0.689	0.584	0.893	0.869	0.729	0.101
Head 4 (Layer 5)	7.207	0.68	8.076	0.510	0.702	0.615	0.899	0.867	0.732	0.101
Head 1 (Layer 6)	7.206	0.681	8.093	0.509	0.684	0.576	0.894	0.867	0.731	0.101
Head 2 (Layer 6)	7.137	0.681	8.188	0.507	0.697	0.600	0.901	0.867	0.731	0.101
Head 3 (Layer 6)	7.144	0.681	8.103	0.508	0.699	0.608	0.901	0.871	0.731	0.101
Head 4 (Layer 6)	7.260	0.681	8.589	0.505	0.692	0.589	0.891	0.844	0.730	0.102

Table S15: Ablation study results for pruning individual heads of the first layer in the multi-task learning model on the SCAN-B DRFi dataset. Cases with the most significant performance degradation upon pruning each head are highlighted in bold. Metrics where higher values indicate better performance are marked with (↑), while lower values are better for metrics marked with (↓). NHG, Nottingham histological grade; DRFi, distant recurrence-free interval; MAE, mean absolute error; SCC, Spearman's correlation coefficient; ACC, accuracy; C-Index, Concordance Index; IBS, Integrated Brier Score; MHSA, multi-head self-attention; GWFF, gene-wise feed-forward.

Pruned layer/head	Age		Tumor Size		NHG		PAM50		DRFi	
	MAE(↓)	SCC(↑)	MAE(↓)	SCC(↑)	ACC(↑)	F1(↑)	ACC(↑)	F1(↑)	C-Index(↑)	IBS(↓)
Original	7.321	0.647	6.716	0.522	0.738	0.664	0.927	0.903	0.784	0.054
Layer 1	30.760	-0.001	29.348	-0.233	0.505	0.224	0.525	0.138	0.634	0.073
Layer 2	11.624	0.579	17.846	0.477	0.527	0.270	0.574	0.258	0.735	0.066
Layer 3	8.536	0.646	7.233	0.442	0.596	0.581	0.647	0.560	0.696	0.065
Layer 4	7.746	0.648	6.544	0.528	0.641	0.588	0.656	0.643	0.708	0.066
Layer 5	7.656	0.658	6.809	0.499	0.684	0.644	0.790	0.723	0.710	0.063
Layer 6	7.966	0.634	6.764	0.498	0.633	0.619	0.808	0.752	0.699	0.065
MHSA (Layer 1)	28.474	0.100	11.954	-0.023	0.331	0.166	0.245	0.079	0.384	0.073
MHSA (Layer 2)	15.984	0.643	11.126	0.433	0.568	0.347	0.534	0.218	0.441	0.071
MHSA (Layer 3)	7.748	0.628	7.295	0.413	0.666	0.644	0.526	0.474	0.682	0.066
MHSA (Layer 4)	7.508	0.642	6.663	0.512	0.717	0.668	0.817	0.789	0.722	0.064
MHSA (Layer 5)	7.647	0.651	6.713	0.501	0.667	0.619	0.833	0.766	0.707	0.063
MHSA (Layer 6)	7.753	0.635	6.800	0.493	0.622	0.606	0.801	0.737	0.716	0.064
GWFF (Layer 1)	33.964	0.340	122.954	0.307	0.509	0.234	0.604	0.271	0.662	0.360
GWFF (Layer 2)	7.553	0.655	7.296	0.428	0.689	0.621	0.423	0.242	0.679	0.064
GWFF (Layer 3)	7.788	0.656	7.048	0.491	0.630	0.626	0.842	0.712	0.721	0.064
GWFF (Layer 4)	7.821	0.653	6.705	0.514	0.687	0.669	0.829	0.783	0.701	0.066
GWFF (Layer 5)	7.612	0.653	6.768	0.497	0.651	0.638	0.881	0.827	0.708	0.064
GWFF (Layer 6)	7.809	0.647	6.735	0.501	0.669	0.654	0.863	0.815	0.688	0.065
Head 1 (Layer 1)	11.660	**0.606**	6.664	0.525	0.693	0.527	0.731	0.623	0.665	0.066
Head 2 (Layer 1)	9.220	0.624	**7.625**	**0.414**	**0.438**	**0.446**	0.732	0.565	0.686	**0.068**
Head 3 (Layer 1)	**16.030**	0.628	7.160	0.521	0.679	0.535	**0.719**	**0.339**	**0.591**	**0.068**
Head 4 (Layer 1)	8.360	0.657	7.508	0.444	0.697	0.599	0.780	0.648	0.680	0.064
Head 1 (Layer 2)	8.284	0.614	7.091	0.475	0.691	0.648	0.627	0.561	0.686	0.064
Head 2 (Layer 2)	7.568	0.658	6.810	0.487	0.544	0.536	0.733	0.646	0.688	0.066
Head 3 (Layer 2)	7.717	0.644	6.707	0.493	0.665	0.645	0.816	0.747	0.699	0.065
Head 4 (Layer 2)	9.707	0.647	6.655	0.513	0.707	0.654	0.849	0.756	0.722	0.064
Head 1 (Layer 3)	7.491	0.646	6.776	0.492	0.687	0.659	0.780	0.725	0.700	0.063
Head 2 (Layer 3)	7.641	0.643	6.745	0.489	0.634	0.618	0.800	0.748	0.688	0.065
Head 3 (Layer 3)	7.475	0.638	6.839	0.476	0.672	0.657	0.886	0.836	0.708	0.064
Head 4 (Layer 3)	7.459	0.647	6.809	0.480	0.629	0.621	0.807	0.754	0.682	0.065
Head 1 (Layer 4)	7.660	0.639	6.695	0.507	0.682	0.664	0.787	0.749	0.698	0.064
Head 2 (Layer 4)	7.535	0.646	6.700	0.498	0.677	0.659	0.862	0.806	0.719	0.064
Head 3 (Layer 4)	7.396	0.648	6.692	0.490	0.669	0.652	0.869	0.806	0.714	0.064
Head 4 (Layer 4)	7.564	0.647	6.677	0.497	0.701	0.679	0.849	0.799	0.711	0.064
Head 1 (Layer 5)	7.393	0.648	6.705	0.500	0.679	0.661	0.862	0.804	0.707	0.064
Head 2 (Layer 5)	7.346	0.649	6.767	0.485	0.665	0.649	0.874	0.819	0.696	0.064
Head 3 (Layer 5)	7.582	0.647	6.749	0.490	0.711	0.665	0.882	0.827	0.714	0.063
Head 4 (Layer 5)	7.441	0.648	6.733	0.490	0.667	0.653	0.832	0.787	0.706	0.064
Head 1 (Layer 6)	7.625	0.647	6.702	0.493	0.625	0.614	0.818	0.756	0.707	0.064
Head 2 (Layer 6)	7.543	0.647	6.715	0.501	0.653	0.637	0.807	0.746	0.728	0.063
Head 3 (Layer 6)	7.684	0.641	6.710	0.498	0.705	0.674	0.883	0.835	0.706	0.064
Head 4 (Layer 6)	7.509	0.648	6.893	0.476	0.616	0.608	0.833	0.773	0.684	0.066

Table S16: Ablation study evaluating the impact of gene embedding initialization on the SCAN-B OS dataset. "Random Init" uses randomly initialized gene embeddings without pretraining. "Random PPI" uses gene embeddings pretrained on a degree-preserving randomized version of the STRING network (constructed via multiple edge swaps while preserving node degrees). Metrics with higher values indicating better performance are marked with (↑), while those with lower values being better are marked with (↓). NHG, Nottingham histological grade; OS, overall survival; MAE, mean absolute error; RMSE, root mean squared error; PCC, Pearson's correlation coefficient; SCC, Spearman's correlation coefficient; C-Index, Concordance Index; IBS, Integrated Brier Score; Protein-Protein Interaction, PPI.

Method		Age				Tumor size			
		MAE (↓)	RMSE(↓)	PCC(↑)	SCC(↑)	MAE (↓)	RMSE(↓)	PCC(↑)	SCC(↑)
Single-task Learning	TxT	7.185±0.194	9.404±0.190	0.697±0.018	0.684±0.025	6.778±0.171	10.535±0.418	0.442±0.025	0.485±0.007
	Random Init	8.095±1.258	10.444±1.251	0.650±0.256	0.646±0.243	7.909±0.171	11.465±0.526	0.379±0.042	0.424±0.069
	Random PPI	8.411±0.162	10.488±0.194	0.630±0.089	0.629±0.086	7.866±0.566	11.248±0.829	0.391±0.138	0.450±0.123
Multi-task Learning	TxT	7.140±0.170	9.238±0.137	0.710±0.013	0.681±0.021	6.721±0.160	10.484±0.421	0.452±0.022	0.519±0.012
	Random Init	7.832±0.133	9.996±0.178	0.660±0.059	0.642±0.059	7.979±0.161	11.490±0.538	0.391±0.070	0.448±0.092
	Random PPI	7.803±0.146	9.985±0.188	0.651±0.049	0.641±0.052	7.982±0.161	11.481±0.531	0.399±0.043	0.444±0.062

Method		NHG				PAM50			
		Accuracy (↑)	Precision (↑)	Recall (↑)	F1 (↑)	Accuracy (↑)	Precision (↑)	Recall (↑)	F1 (↑)
Single-task Learning	TxT	0.715±0.011	0.752±0.026	0.637±0.014	0.649±0.011	0.920±0.007	0.904±0.019	0.892±0.017	0.891±0.013
	Random Init	0.685±0.103	0.729±0.261	0.582±0.142	0.649±0.203	0.888±0.161	0.864±0.327	0.856±0.283	0.856±0.310
	Random PPI	0.685±0.067	0.705±0.174	0.587±0.089	0.603±0.129	0.881±0.015	0.883±0.022	0.851±0.028	0.864±0.020
Multi-task Learning	TxT	0.722±0.006	0.736±0.018	0.675±0.016	0.667±0.013	0.920±0.007	0.907±0.011	0.892±0.018	0.891±0.010
	Random Init	0.692±0.007	0.727±0.002	0.613±0.000	0.623±0.002	0.893±0.010	0.884±0.002	0.870±0.000	0.867±0.002
	Random PPI	0.692±0.007	0.727±0.002	0.613±0.000	0.623±0.002	0.893±0.010	0.884±0.002	0.870±0.000	0.867±0.002

Method		OS	
		C-Index (↑)	IBS (↓)
Single-task Learning	TxT	0.701±0.014	0.087±0.004
	Random Init	0.651±0.039	0.100±0.005
	Random PPI	0.652±0.092	0.100±0.005
Multi-task Learning	TxT	0.797±0.017	0.084±0.007
	Random Init	0.704±0.059	0.099±0.004
	Random PPI	0.700±0.092	0.099±0.004

Table S17: Ablation study evaluating the impact of gene embedding initialization on the SCAN-B DRFi dataset. “Random Init” uses randomly initialized gene embeddings without pretraining. “Random PPI” uses gene embeddings pretrained on a degree-preserving randomized version of the STRING network (constructed via multiple edge swaps while preserving node degrees). Metrics with higher values indicating better performance are marked with (↑), while those with lower values being better are marked with (↓). NHG, Nottingham histological grade; DRFi, distant recurrence-free interval; MAE, mean absolute error; RMSE, root mean squared error; PCC, Pearson's correlation coefficient; SCC, Spearman's correlation coefficient; C-Index, Concordance Index; IBS, Integrated Brier Score; Protein-Protein Interaction, PPI.

Method		Age				Tumor size			
		MAE (↓)	RMSE(↓)	PCC(↑)	SCC(↑)	MAE (↓)	RMSE(↓)	PCC(↑)	SCC(↑)
Single-task Learning	TxT	7.349±0.128	9.522±0.208	0.711±0.020	0.679±0.015	6.920±0.221	10.801±0.630	0.399±0.020	0.439±0.029
	Random Init	8.463±0.331	10.058±0.292	0.634±0.095	0.628±0.093	7.950±0.276	11.300±0.704	0.323±0.134	0.359±0.132
	Random PPI	8.425±0.330	10.039±0.301	0.631±0.069	0.624±0.070	7.654±0.594	11.886±0.916	0.323±0.162	0.352±0.159
Multi-task Learning	TxT	7.328±0.121	9.410±0.248	0.720±0.017	0.684±0.017	6.717±0.216	10.268±0.743	0.451±0.023	0.521±0.022
	Random Init	7.900±0.907	9.909±0.927	0.651±0.186	0.645±0.178	7.938±0.420	11.352±0.856	0.352±0.131	0.395±0.146
	Random PPI	7.973±0.250	9.987±0.266	0.662±0.065	0.652±0.055	7.424±0.234	11.439±0.736	0.356±0.099	0.397±0.142

Method		NHG				PAM50			
		Accuracy (↑)	Precision (↑)	Recall (↑)	F1 (↑)	Accuracy (↑)	Precision (↑)	Recall (↑)	F1 (↑)
Single-task Learning	TxT	0.720±0.010	0.741±0.022	0.638±0.021	0.661±0.011	0.923±0.011	0.915±0.012	0.901±0.017	0.903±0.012
	Random Init	0.682±0.008	0.707±0.003	0.583±0.000	0.623±0.002	0.882±0.010	0.873±0.021	0.852±0.021	0.851±0.017
	Random PPI	0.690±0.069	0.709±0.171	0.587±0.090	0.607±0.130	0.899±0.014	0.875±0.023	0.859±0.033	0.864±0.024
Multi-task Learning	TxT	0.735±0.019	0.743±0.021	0.657±0.013	0.667±0.020	0.922±0.012	0.910±0.009	0.904±0.014	0.907±0.017
	Random Init	0.699±0.057	0.716±0.154	0.614±0.097	0.625±0.133	0.890±0.107	0.878±0.237	0.856±0.176	0.862±0.209
	Random PPI	0.692±0.008	0.717±0.003	0.613±0.000	0.623±0.002	0.898±0.012	0.884±0.002	0.870±0.000	0.866±0.002

Method		DRFi	
		C-Index (↑)	IBS (↓)
Single-task Learning	TxT	0.718±0.062	0.059±0.004
	Random Init	0.651±0.055	0.064±0.005
	Random PPI	0.656±0.154	0.065±0.004
Multi-task Learning	TxT	0.787±0.062	0.055±0.006
	Random Init	0.706±0.121	0.064±0.005
	Random PPI	0.735±0.130	0.064±0.005

References

[1] Stephane Fotso. Deep neural networks for survival analysis based on a multi-task framework. *arXiv preprint arXiv:1801.05512*, 2018.

[2] Damian Szklarczyk, Rebecca Kirsch, Mikaela Koutrouli, Katerina Nastou, Farrokh Mehryary, Radja Hachilif, Annika L Gable, Tao Fang, Nadezhda T Doncheva, Sampo Pyysalo, et al. The string database in 2023: protein–protein association networks and functional enrichment analyses for any sequenced genome of interest. *Nucleic acids research*, 51(D1):D638–D646, 2023.

[3] Johan Staaf, Jari Häkkinen, Cecilia Hegardt, Lao H Saal, Siker Kimbung, Ingrid Hedenfalk, Tonje Lien, Therese Sørlie, Bjørn Naume, Hege Russnes, et al. Rna sequencing-based single sample predictors of molecular subtype and risk of recurrence for clinical assessment of early-stage breast cancer. *NPJ breast cancer*, 8(1):94, 2022.

[4] Jason E Farrar, Heather L Schuback, Rhonda E Ries, Daniel Wai, Oliver A Hampton, Lisa R Trevino, Todd A Alonzo, Jaime M Guidry Auvil, Tanja M Davidsen, Patee Gesuwan, et al. Genomic profiling of pediatric acute myeloid leukemia reveals a changing mutational landscape from disease diagnosis to relapse. *Cancer research*, 76(8):2197–2205, 2016.

[5] Brigham & Women's Hospital & Harvard Medical School Chin Lynda 9 11 Park Peter J. 12 Kucherlapati Raju 13, Genome data analysis: Baylor College of Medicine Creighton Chad J. 22 23 Donehower Lawrence A. 22 23 24 25, Institute for Systems Biology Reynolds Sheila 31 Kreisberg Richard B. 31 Bernard Brady 31 Bressler Ryan 31 Erkkila Timo 32 Lin Jake 31 Thorsson Vesteinn 31 Zhang Wei 33 Shmulevich Ilya 31, et al. Comprehensive molecular portraits of human breast tumours. *Nature*, 490(7418):61–70, 2012.

[6] Ashton C Berger, Anil Korkut, Rupa S Kanchi, Apurva M Hegde, Walter Lenoir, Wenbin Liu, Yuexin Liu, Huihui Fan, Hui Shen, Visweswaran Ravikumar, et al. A comprehensive pan-cancer molecular study of gynecologic and breast cancers. *Cancer cell*, 33(4):690–705, 2018.

[7] Arthur Liberzon, Chet Birger, Helga Thorvaldsdóttir, Mahmoud Ghandi, Jill P Mesirov, and Pablo Tamayo. The molecular signatures database hallmark gene set collection. *Cell systems*, 1(6):417–425, 2015.

[8] Aditya Grover and Jure Leskovec. node2vec: Scalable feature learning for networks. In *Proceedings of the 22nd ACM SIGKDD international conference on Knowledge discovery and data mining*, pages 855–864, 2016.

[9] Ashish Vaswani, Noam Shazeer, Niki Parmar, Jakob Uszkoreit, Llion Jones, Aidan N Gomez, Łukasz Kaiser, and Illia Polosukhin. Attention is all you need. *Advances in neural information processing systems*, 30, 2017.
